# A Green, Solvent- and Cation-Free Approach for Preparing 5-Fluorouracil-Loaded Alginate Nanoparticles Using Microfluidic Technology

**DOI:** 10.3390/pharmaceutics17040438

**Published:** 2025-03-29

**Authors:** Abdolelah Jaradat, Ali Alazzo, Mohammad F. Bayan, Wasfy Obeidat

**Affiliations:** 1 Department of Applied Pharmaceutical Sciences and Clinical Pharmacy, Faculty of Pharmacy, Isra University, Amman 11622, Jordan; 2 Department of Pharmaceutics, College of Pharmacy, University of Mosul, Mosul 41002, Iraq; alialazzo@uomosul.edu.iq; 3 Faculty of Pharmacy, Philadelphia University, P.O. Box 1, Amman 19392, Jordan; mbayan@philadelphia.edu.jo; 4 Department of Pharmaceutical Technology, Faculty of Pharmacy, Jordan University of Science and Technology, P.O. Box 3030, Irbid 22110, Jordan; obeidatw@just.edu.jo

**Keywords:** alginate nanoparticles, 5-FU, microfluidic technology, proton gelation, ecologically friendly, optimum formula

## Abstract

**Background/Objectives**: Alginate nanoparticles (NPs) are commonly synthesised using either an emulsion technique that involves organic solvent use or ionotropic gelation utilising multivalent cations, e.g., Ca^+2^. However, the extensive use of organic solvents imposes detrimental effects on the ecosystem, and using multivalent cations as crosslinkers could eventually lead to the leakage of these cations, thus disrupting nanoparticle matrices. Therefore, this study aimed to overcome the limitations of these techniques by eliminating the usage of organic solvents and multivalent cations. **Methods**: In this research, alginate nanoparticles were synthesised using proton gelation by microfluidic technology through protonating alginate carboxylate groups to crosslink alginate chains through H-bond formation. **Results**: The prepared acid-gelled alginate nanoparticles demonstrated an MHD circa 200 nm and a PDI of less than 0.4 at pH 0.75. Moreover, 5-FU was successfully encapsulated into acid-gelled alginate nanoparticles and displayed a high EE% of around 30%, comparable to the EE% at high alginate concentration and molecular weight (0.4 H-ALG) achieved by Ca^+2^-crosslinked alginate nanoparticles; however, 5-FU NPs had superior characteristics, i.e., a lower MHD (around 500 nm) and PDI (<0.5). The optimum formula (0.4 H-ALG) was explored at various pH values, i.e., low pH of 4.5 and high pH of 10, and alginate NPs produced by acid gelation demonstrated high stability in terms of MHD and PDI, with slight changes at different pH values, indicating stable crosslinking of alginate matrices prepared by technology compared with Ca^+2^-crosslinked alginate NPs. **Conclusions**: In conclusion, this research has invented an ecologically friendly approach to producing acid-gelled alginate nanoparticles with superior characteristics compared with the conventional methods, and they could be harnessed as nanocarriers for therapeutics delivery (5-FU). Also, this research offers a promising approach for developing eco-friendly and biocompatible drug carriers. The produced nanoparticles have the potential to enhance drug stability, improve controlled release, and minimise toxic effects, making them suitable for pharmaceutical applications.

## 1. Introduction

Nanotechnology has been extensively used for biological and medical applications [1,2]. For instance, nanoparticles made out of different kinds of biomaterials, polymers, natural polysaccharide and/or inorganic materials have been widely used for the delivery of small drug molecules [3], as well as biomacromolecules such as proteins and RNA [4]. Nanoparticle-based systems have also been utilised for diagnostic processes by employing various stimuli-responsive nanosensors [5,6]. However, before using these nanocarriers for drug delivery applications, their safety concerns must be carefully addressed. Nanoparticle biodegradability in the cellular environment is considered a major limitation regarding their use, as they may cause severe toxicity by accumulating inside the cells and leading to intracellular changes [7]. In this sense, both the toxicity and biodegradability of polymeric nanoparticles have been previously monitored [8,9]. Therefore, natural biocompatible and biodegradable polymers such as alginate, chitosan, and pectin have been employed for the production of nanoparticles [10,11,12,13,14,15]. Specifically, alginate has been extensively studied as a drug carrier owing to its biocompatibility, availability, and environmentally friendly and biological safety properties. For instance, alginate nanoparticles have not produced any systemic toxicity, as demonstrated by extensive studies over the last few decades [16]. On the other hand, the only toxicity concerns in relation to using alginate particles stems from its polymer chain crosslinking process, where it has been found that physical crosslinking using multivalent cations, e.g., Ca^+2^, is preferred over chemical crosslinking [17]. Moreover, controlling the degradation of the drug carrier composed of alginate is vital for drug delivery and diagnostic procedures in physiological conditions. Diverse strategies aimed to achieve kidney clearance of alginate carriers by maintaining a molecular weight of alginate below 50 kDa [18]. For instance, hydrogels comprising a blend of high and partially oxidised low molecular weight alginate demonstrated accelerated degradation within 7 days, with nearly complete mass loss after 40 days [19]. Alginate nanoparticles could be employed for drug delivery, including encapsulation of the drugs, allowing for controlled release and targeted delivery to specific sites in the body. They are particularly useful in delivering poorly soluble drugs, which improves bioavailability and therapeutic efficacy. Alginate nanoparticles can serve as adjuvants or carriers for vaccines, enhancing immune responses and stability. They can improve the delivery of antigens to immune cells, leading to better vaccination outcomes. Additionally, they can be used to transport genetic material, such as DNA or RNA, into cells. Their biocompatibility and ability to form stable complexes with nucleic acids make them suitable for gene therapy applications. Moreover, alginate nanoparticles can be utilised in tissue engineering scaffolds to promote cell adhesion, proliferation, and differentiation. They can deliver growth factors or drugs to enhance tissue regeneration.

Despite the numerous advantages of alginate-based nanoparticles, the production of these tiny particles requires multiple processes [20,21]. A myriad of methods for the synthesis of alginate nanoparticles have been utilised, such as using a nanoprecipitation method by the addition of organic solvent, such as an acetone and ethanol alginate solution [22]; preparing a nano-emulsion using organic-aqueous phase stabilisation and subsequent solvent evaporation [23,24]; or an ionotropic gelation method, which relies on the crosslinking of alginate using multivalent cations such as calcium and aluminium [25,26,27]. However, the consumption of large quantities of organic solvents, such as acetone and ethanol, has a detrimental impact on the ecological system. Moreover, the primary drawback of ionotropic gelation used for alginate nanoparticle synthesis is the release of crosslinking ions, leading to a weakening of the formed crosslinked matrices of alginate nanoparticles and their eventual dissolution [28]. The same finding has also been reported elsewhere, as the exchange of calcium ions with sodium ions and the eventual leakage of calcium ions out of the alginate matrices forming alginate beads has led to the loss of the structural integrity of alginate matrices and the subsequent degradation of the calcium beads [29,30]. The process of multivalent cation leakage from the crosslinked alginate matrices has also been reported by some researchers to be accelerated in the presence of chelating agents such as Phosphate, EDTA, and citrate or high concentrations of competing cations, e.g., sodium and potassium [31,32]. The leakage of trivalent cations such as ferrous from alginate matrices has also been reported, although stronger alginate matrices were formed in the presence of Fe^3+^ in comparison with those formed by Ca^+2^ crosslinking [33,34]. A research study has found that a Na-alginate solution has the capability, under specific conditions, of experiencing sol-gel transformation at a pH lower than the pKa for uronic acid residues. These 3D networks of the formed alginate gel are thought to be a result of alginate chain crosslinking/entanglement, and they are likely upheld by intermolecular hydrogen bonding. This study also outlined the impact of various alginate characteristics, including chemical composition, sequence, and molecular weight, on both mechanical properties and the intricate structure of the network. The findings of this study demonstrated that alginic acid gels exhibited similarities to ionically crosslinked alginate gels, as guluronic acid blocks emerge as the primary constituents crucial for gel formation [35]. Collectively, it is essential to highlight the limitations of conventional nanoparticle synthesis methods, including the use of toxic chemicals, complex procedures, high energy consumption, and environmental concerns. These challenges affect biocompatibility, scalability, and sustainability, limiting their effectiveness in drug delivery applications. To provide a clearer comparison, Table 1 outlines the key differences between ionotropic gelation, acid gelation, and the emulsion method using an organic solvent, emphasising their principles, advantages, and limitations.

Moreover, microfluidic technology is widely recognised as superior to conventional bulk mixing techniques, e.g., batch synthesis methods for producing nanoparticles [36]. This is primarily due to their enhanced control over the preparation process, resulting in greater reproducibility and more consistent nanoparticle properties [37]. Microfluidic devices operate under continuous flow conditions, enabling the efficient and controllable mixing of solutions [38]. This results in a homogeneous reaction environment due to the intimate mixing of reactants in a narrow, well-defined space of the microchannels [39]. The small dimensions of microfluidic channels allow for superior temperature control and more efficient heat transfer, which is critical in maintaining uniform reaction conditions [38]. Also, in microfluidic systems, the reagents can be introduced at specific time intervals during the reaction process, allowing precise temporal control over the synthesis of nanoparticles. It provides the ability to control process kinetics and enables fine-tuning of nanoparticle properties such as size, distribution, and morphology [40,41,42]. In summary, while batch synthesis methods have been widely used for many years, microfluidic technology offers distinct advantages that can lead to improved efficiency, quality, and safety in nanoparticle synthesis and other applications. These benefits position microfluidics as a promising alternative for researchers seeking to develop more sustainable and effective synthesis methods.

**Figure 1:**
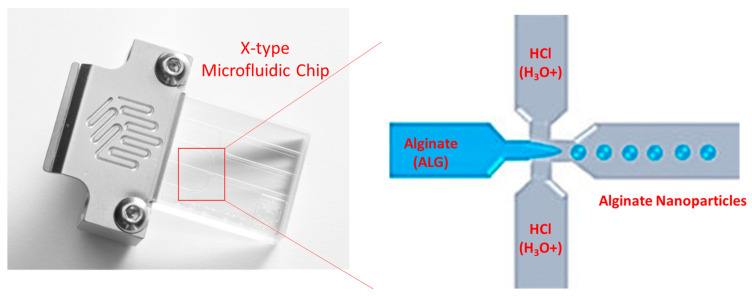
Schematic diagram showing the proposed preparation method of acid-gelled alginate nanoparticles produced by proton gelation of alginate chains using an X-type microfluidic chip, i.e., lab-on-a-chip technology.

In light of that, the current study was dedicated to generating a novel method to produce alginate nanoparticles relying on the sol-gel transformation of alginate chains upon the addition of hydronium ions brought by acidifying an aqueous solution as a gelling agent using a hydrodynamic flow focusing technique provided by X-type microfluidic technology, as displayed in the schematic diagram in Figure 1. Therefore, this research aimed to synthesise alginate nanoparticles using an entirely green, ecologically friendly method that is completely devoid of the consumption of organic solvents, e.g., acetone, ethanol, or dimethyl sulfoxide. The new technology relies on employing diluted acid as a source of protons for gelling and crosslinking the alginate polymeric chains to produce alginate nanoparticles by harnessing a high-precision technique, which is lab-on-a-chip technology, i.e., microfluidics.

## 2. Impact of the Study on Sustainability

This research is significant as it explores the innovative use of alginate nanoparticles, highlighting their potential to foster sustainability and promote eco-friendly practices across various industries. As a natural polymer derived from brown algae, alginate offers a biodegradable and non-toxic alternative to synthetic materials, addressing the growing concerns surrounding environmental pollution and resource depletion. In a broader perspective, the proposed method could be used as an alternative technology to the utilisation of the multivalent cation crosslinking method or the organic solvent method for producing alginate nanoparticles/nanogels, as they entail various disadvantages, as described earlier. Moreover, the sustainable development goals (SDGs) set by the UN, in particular, goal number 3, which states “ensure healthy lives and promote well-being for all at all ages”, where the goal is focused on reducing illnesses and death from hazardous chemicals and pollution.

## 3. Materials and Methods

### 3.1. Materials and Equipment

Alginic acid sodium salt low viscosity (ALG) was supplied by Sigma-Aldrich, Burlington, MA, USA. Glacial acetic acid and sodium hydroxide were supplied by Az Chem, Nanjing, Jiangsu, China, whereas calcium chloride and disodium hydrogen phosphate were supplied by Ghtech, Panyu, Guangzhou, China. Hydrochloric acid (HCl) 33–36% was supplied by BBC Chemicals, Las Vegas, NV, USA. Sodium acetate trihydrate was supplied by Central Drug House Ltd., New Delhi, Delhi 110002, India. Phosphate-buffered saline (PBS) was purchased from Thermo Fisher Scientific, Waltham, MA, USA. Except when otherwise noted, all of the substances used in this experiment were of analytical grade.

### 3.2. Synthesis of Proton-Gelled Alginate Nanoparticles

Alginate nanoparticles were prepared by the newly invented method using protons/hydronium ions brought by the acidification of the aqueous phase using diluted hydrochloric acid (HCl) either before the addition of the chemotherapeutic drug 5-fluorouracil (5-FU), i.e., blank alginate nanoparticles, or after adding the chemotherapeutic drug 5-fluorouracil (5-FU), i.e., 5-FU-loaded alginate nanoparticles. An American NE-300 Infusion ONE Syringe Pump (Darwin Microfluidics, West Lafayette, IN, USA) and a British Dolomite Large Droplet Junction microfluidic chip (Dolomite Microfluidics, Royston, Hertfordshire, UK) made up the system utilised to create nanoparticles. The microfluidic chip was observed using a Nikon Alphaphot YS lens from Nikon Corporation, Shinagawa-ku, Tokyo, Japan.

### 3.3. Synthesis of Unloaded Alginate Nanoparticles (Blank Nanoparticles)

#### 3.3.1. Synthesis of Blank Alginate Nanoparticles Made of Low-Viscosity and Low-Concentration Alginate Polymer

**Figure 2:**
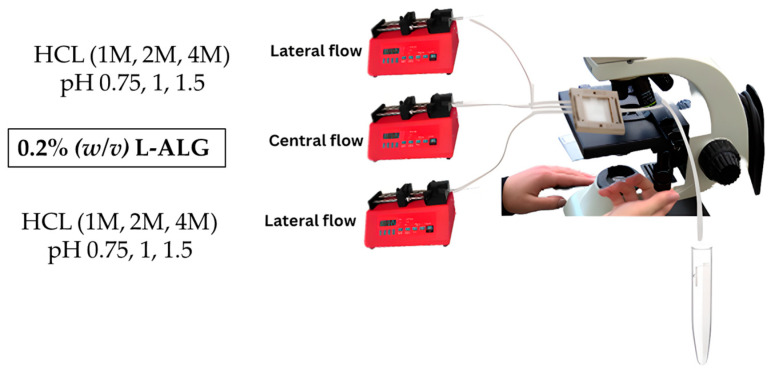
Synthesis of blank alginate nanoparticles made of low-viscosity and low-concentration alginate polymer.

A low-viscosity alginate solution with a final concentration of 0.2 g/100 mL (0.2% (*w*/*v*)) was prepared and placed in the central flow compartment/line and was run at a specified flow rate of 0.25 mL/min. On the other hand, different molar concentrations of hydrochloric acid (HCL) were prepared, which were 1, 2, and 4 M, and placed in the lateral flow line with a specific flow rate of 0.5 mL/min and left to run to produce drug-free alginate nanoparticles, which are denoted as (0.2 L-ALG) as illustrated in Figure 2.

#### 3.3.2. Synthesis of Blank Alginate Nanoparticles Made of High-Viscosity and Low-Concentration Alginate Polymer

**Figure 3:**
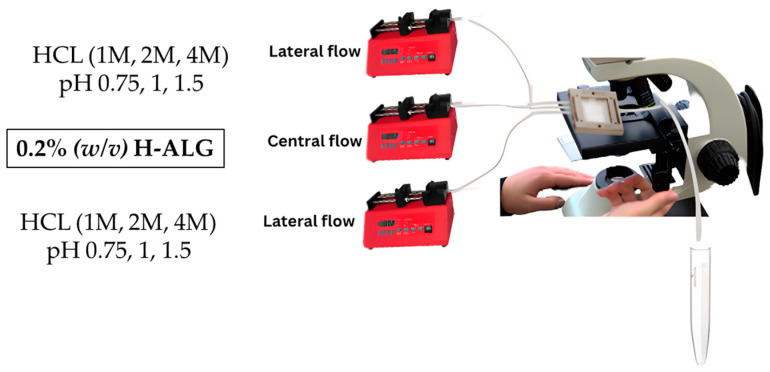
Synthesis of blank alginate nanoparticles made of high-viscosity and low-concentration alginate polymer.

A high-viscosity alginate solution with a final concentration of 0.2 g/100 mL (0.2% (*w*/*v*)) was prepared and placed in the central flow compartment/line and was run at a specified flow rate of 0.25 mL/min. On the other hand, different molar concentrations of hydrochloric acid were prepared, which were 1, 2, and 4 M, and placed in the lateral flow line with a specific rate of 0.5 mL/min and left to run to produce drug-free alginate nanoparticles, which are denoted as (0.2 H-ALG) as illustrated in Figure 3.

#### 3.3.3. Synthesis of Blank Alginate Nanoparticles Made of Low-Viscosity and High-Concentration Alginate Polymer

**Figure 4:**
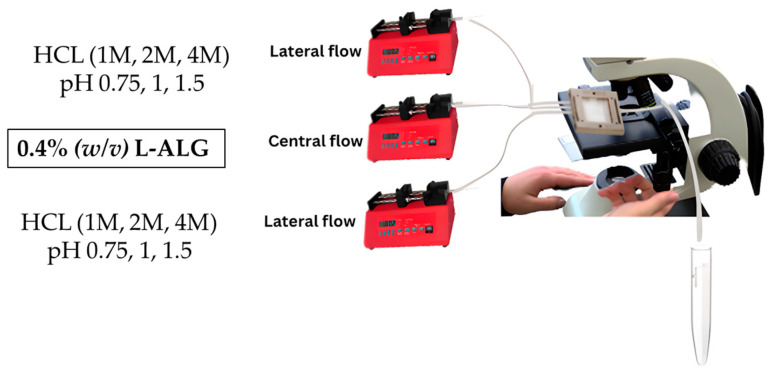
Synthesis of blank alginate nanoparticles made of low-viscosity and high-concentration alginate polymer.

A low-viscosity alginate solution with a final concentration of 0.4 g/100 mL (0.4% (*w*/*v*)) was placed in the central flow compartment/line and was run at a specified flow rate of 0.25 mL/min. On the other hand, different molar concentrations of hydrochloric acid (HCl) were prepared, which were 1, 2, and 4 M, and placed in the lateral flow line with a specific rate of 0.5 mL/min and left to run to produce drug-free alginate nanoparticles, which are denoted as (0.4 L-ALG) as illustrated in Figure 4.

#### 3.3.4. Synthesis of Blank Alginate Nanoparticles Made of High-Viscosity and High-Concentration Alginate Polymer

**Figure 5:**
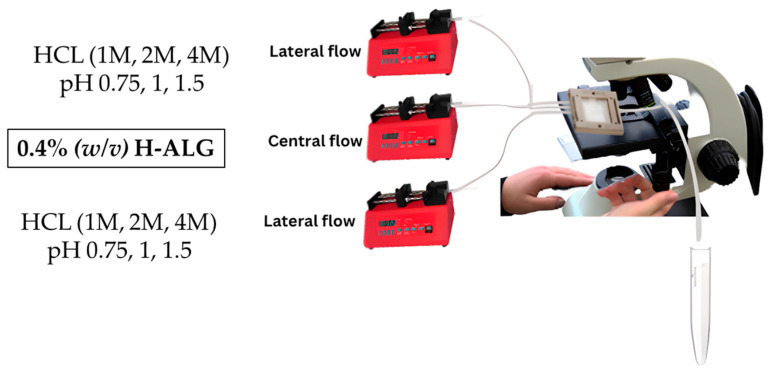
Synthesis of blank alginate nanoparticles made of high-viscosity and high-concentration alginate polymer.

A high-viscosity alginate solution with a final concentration of 0.4 g/100 mL (0.4% (*w*/*v*)) was placed in the central flow compartment/line and was run at a specified flow rate of 0.25 mL/min. On the other hand, different molar concentrations of hydrochloric acid were prepared, which were 1, 2, and 4 M, and placed in the lateral flow line with a specific rate of 0.5 mL/min and left to run to produce drug-free alginate nanoparticles, which are denoted as (0.4 H-ALG) as illustrated in Figure 5.

### 3.4. Synthesis of Drug-Loaded Alginate Nanoparticles

#### 3.4.1. Synthesis of 5-FU-Loaded Alginate Nanoparticles Made of Low-Viscosity and Low-Concentration Alginate Polymer at High Theoretical Loading

**Figure 6:**
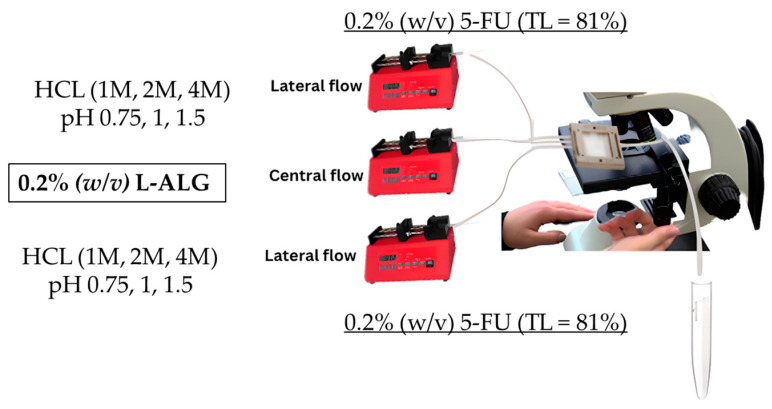
Synthesis of 5-FU-loaded alginate nanoparticles made of low-viscosity and low-concentration alginate polymer at high theoretical loading.

To produce 5-FU-loaded low-viscosity alginate nanoparticles at a theoretical loading of 81% (TL% = 81)) while using HCl/proton as a gelling agent, the low-viscosity alginate solution with a final concentration of 0.2 g/100 mL (0.2% (*w*/*v*)) was placed in the central flow compartment/line and was run at a specified flow rate of 0.25 mL/min. On the other hand, different molar concentrations of hydrochloric acid (HCl) were prepared, which were 1, 2, and 4 M. After that, 5-FU was dissolved in the HCl solutions to provide a 5-FU solution with a final concentration of 0.2 g/100 mL (0.2% (*w*/*v*)), which was then placed in the lateral flow line and left to run at a specific rate of 0.5 mL/min to produce 5-FU-loaded alginate nanoparticles, which are denoted as (0.2 L-ALG (TL% = 81)) as illustrated in Figure 6.

#### 3.4.2. Synthesis of 5-FU-Loaded Alginate Nanoparticles Made of High-Viscosity and Low-Concentration Alginate Polymer at High Theoretical Loading

**Figure 7:**
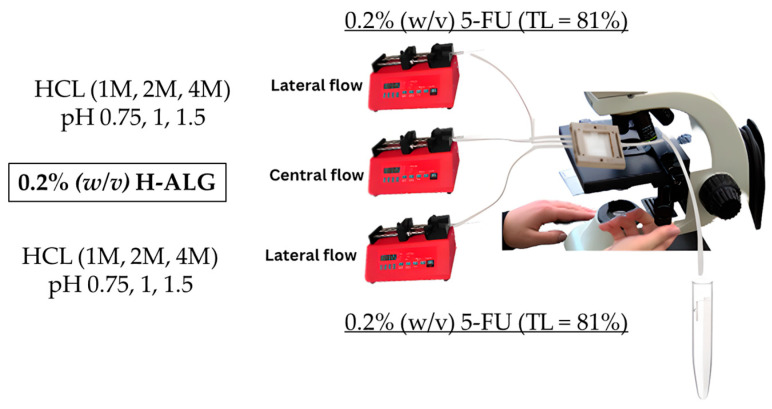
Synthesis of 5-FU-loaded alginate nanoparticles made of high-viscosity and low-concentration alginate polymer at high theoretical loading.

To produce 5-FU-loaded high-viscosity alginate nanoparticles at a theoretical loading of 81% (TL% = 81)) while using HCl/proton as a gelling agent, the high-viscosity alginate solution with a final concentration of 0.2 g/100 mL (0.2% (*w*/*v*)) was placed in the central flow compartment/line with a specified flow rate of 0.25 mL/min. On the other hand, different molar concentrations of hydrochloric acid were prepared, which were 1, 2, and 4 M. After that, 5-FU was dissolved in the HCl solutions to provide a 5-FU solution with a final concentration of 0.2 g/100 mL (0.2% (*w*/*v*)), which was then placed in the lateral flow line and left to run at a specific rate of 0.5 mL/min to produce 5-FU-loaded alginate nanoparticles, which are denoted as (0.2 H-ALG (TL% = 81)) as illustrated in Figure 7.

#### 3.4.3. Synthesis of 5-FU-Loaded Alginate Nanoparticles Made of Low-Viscosity and High-Concentration Alginate Polymer at Low Theoretical Loading

**Figure 8:**
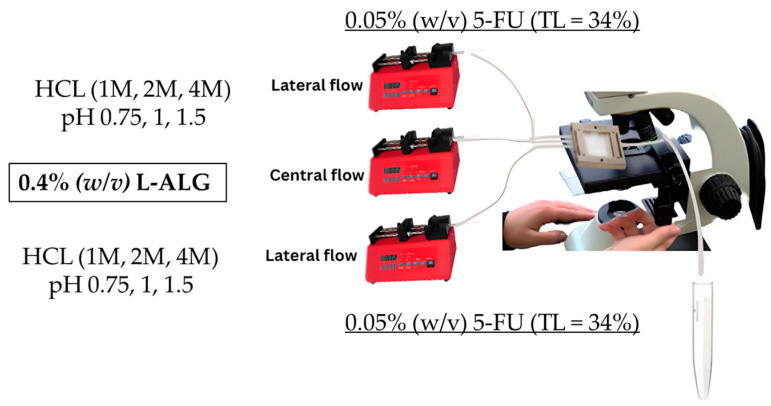
Synthesis of 5-FU-loaded alginate nanoparticles made of low-viscosity and high-concentration alginate polymer at low theoretical loading.

To produce 5-FU-loaded low-viscosity alginate nanoparticles at a theoretical loading of 34% (TL% = 34) while using HCl/proton as a gelling agent, the low-viscosity alginate solution with a final concentration of 0.4 g/100 mL (0.4% (*w*/*v*)) was placed in the central flow compartment/line with a specified flow rate of 0.25 mL/min. On the other hand, different molar concentrations of hydrochloric acid were prepared, which were 1, 2, and 4 M. After that, 5-FU was dissolved in the HCl solutions to provide a 5-FU solution with a final concentration of 0.05 g/100 mL (0.05% (*w*/*v*)), which was then placed in the lateral flow line and left to run at a specific rate of 0.5 mL/min to produce 5-FU-loaded alginate nanoparticles, which are denoted as (0.2 L-ALG (TL% = 34)) as illustrated in Figure 8.

#### 3.4.4. Synthesis of 5-FU-Loaded Alginate Nanoparticles Made of High-Viscosity and High-Concentration Alginate Polymer at Low Theoretical Loading

**Figure 9:**
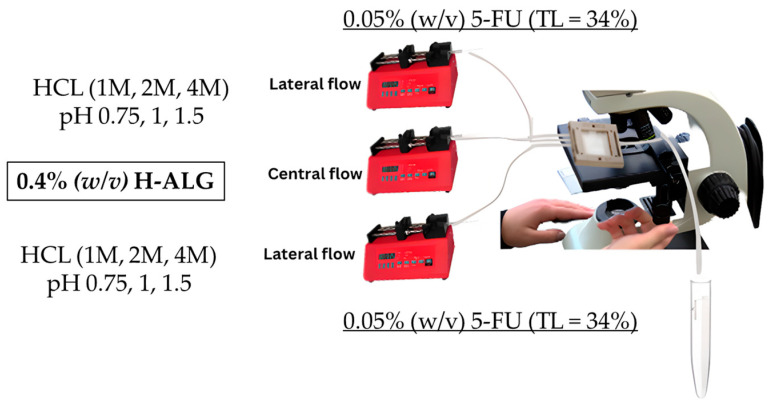
Synthesis of 5-FU-loaded alginate nanoparticles made of high-viscosity and high-concentration alginate polymer at low theoretical loading.

To produce high-viscosity alginate nanoparticles with a theoretical loading of 34% for 5-FU (TL% = 34)) while using HCl/proton as a gelling agent, the high-viscosity alginate solution with a final concentration of 0.4 g/100 mL (0.4% (*w*/*v*)) was placed in the central flow compartment/line with a specified flow rate of 0.25 mL/min. On the other hand, different molar concentrations of hydrochloric acid were prepared, which were 1, 2, and 4 M. After that, 5-FU was dissolved in the HCl solutions to provide a 5-FU solution with a final concentration of 0.05 g/100 mL (0.05% (*w*/*v*)), which was then placed in the lateral flow line and left to run at a specific rate of 0.5 mL/min to produce 5-FU-loaded alginate nanoparticles, which are denoted as (0.4 L-ALG (TL% = 34)) as illustrated in Figure 9.

### 3.5. Synthesis of Drug-Loaded Calcium-Crosslinked Alginate Nanoparticles

#### 3.5.1. Synthesis of 5-FU-Loaded Calcium-Crosslinked Alginate Nanoparticles Prepared at Low Concentration of Alginate Polymer and High Theoretical Loading

To produce 5-FU-loaded alginate nanoparticles crosslinked with calcium chloride at a theoretical loading of 81% (TL% = 81) for 5-FU while using CaCl2 as a crosslinker, two alginate solutions with the same final concentration of 0.2 g/100 mL (0.2% (*w*/*v*)) dissolved in PBS at pH 7.4 and different viscosity levels, either low or high molecular weight grade, were separately placed in the central flow compartment/line with a specified flow rate of 0.25 mL/min. On the other hand, 5-FU was dissolved in a 10 mM CaCl2 solution to provide a 5-FU solution with a final concentration of 0.2 g/100 mL (0.2% (*w*/*v*)), which was placed in the lateral flow lines at a specific rate of 0.5 mL/min to produce calcium-crosslinked alginate nanoparticles loaded with 5-FU using low- or high-viscosity alginate.

#### 3.5.2. Synthesis of 5-FU-Loaded Calcium-Crosslinked Alginate Nanoparticles Prepared at High Concentration of Alginate Polymer and Low Theoretical Loading

To produce 5-FU-loaded alginate nanoparticles crosslinked with calcium chloride at a theoretical loading of 34% for 5-FU while using CaCl2 as a crosslinker, two alginate solutions with the same concentration of 0.4 g/100 mL (0.4% (*w*/*v*)) and different viscosity levels, either low- or high-viscosity grade, were separately placed in the central flow compartment/line with a specified flow rate of 0.25 mL/min. On the other hand, 5-FU was dissolved in a 10 mM CaCl2 solution to provide a 5-FU solution with a final concentration of 0.05 g/100 mL (0.05% (*w*/*v*)), which was placed in the lateral flow line with a specific rate of 0.5 mL/min to produce calcium-crosslinked alginate nanoparticles loaded with 5-FU using low- or high-viscosity alginate.

### 3.6. Characterisation of Acid-Gelled and Calcium-Crosslinked Alginate Nanoparticles

To identify the polydispersity index (PDI), average particle diameter, and zeta potential of the prepared nanoparticles, the following procedure was used: a 0.1 mg/mL of alginate nanoparticle suspension was placed in a 0.1 N sodium chloride medium or an acetate buffer at pH 5. After that, 1 mL of the suspended nanoparticles was placed in a glass cuvette, and the PDI, size distribution, and zeta potential of the suspended nanoparticles were measured using the dynamic light scattering technique (Zetasizer-Advance Pro, Malvern Panalytical Zetasizer Ultra, Malvern, UK). The Sonorex Digitec from BANDELIN electronic, Berlin, Germany, was utilised for sonication.

### 3.7. Measuring the Encapsulation Efficiency of 5-FU

5-FU and calibration curve:

To prepare the 5-FU solution, 0.01 g of 5-FU was dissolved in 10 mL of acetate buffer (pH 3.5). After that, the stock solution was diluted using an acetate buffer at a 1:10 ratio. By reading the absorbance at a wavelength of 266 nm for seven different drug solutions with concentrations of 1.25, 2.5, 5, 7.5, 10, 15, and 20 µg/mL, it was possible to obtain a calibration equation, which was

Absorbance (Abs) = 0.0545 * Concentration (Conc) + 0.0083(1)

Measuring the encapsulation efficiency of 5-FU into alginate nanoparticles using the indirect method:

The encapsulation efficiency percentage (EE%) was determined indirectly by measuring the amount of the unbound drug, and EE% was obtained using the following rule:

Encapsulation efficiency (EE%) = ((Total amount of drug added − Unbound drug))/((Total amount of drug added)) × 100%(2)

After encapsulation, the amount of the unbound hydrophilic drug 5-fluorouracil (5-FU) was determined by centrifuging the drug-loaded nanoparticles at 5000 rpm for 1 h with cooling. The supernatant was collected, and its absorbance was recorded using a UV–visible spectrophotometer. Based on the flow rate of the phase in which the drug was dissolved, it was possible to calculate the total amount of drug added. The Labofuge 200 from Heraeus Sepatech, Potsdam, Germany, and the Amicon^®^ Ultra-15 Centrifugal Filter Unit from Merck KGaA, Darmstadt, Germany, were employed for nanoparticle precipitation.

For drug delivery, we know that the theoretical drug loading is very important for determining the amount of the encapsulated drug relative to the matrix material required for administration. In this project, different values of the theoretical loading (34% and 81%) were used at each polymer concentration. The following theoretical loading (TL%) equation was used to determine the amount of drug added:

Theoretical loading (TL%) = ((Weight of drug))/((Weight of drug + weight of polymer)) × 100%(3)

Then, 2 min later, the nanoparticles were collected, and the supernatant was separated. The supernatant was diluted 160 times with a 3.5 pH acetate buffer for measuring the absorbance, which was used to calculate the EE% by using the calibration equation and uploading the results to an Excel sheet.

### 3.8. Stability of 5-FU-Loaded Alginate Nanoparticles at Different pH Values

To determine the stability of 5-FU-loaded alginate nanoparticles at different pH values, the physical characteristics such as size, polydispersity index, and zeta potential for the alginate acid-precipitated nanoparticles and calcium-crosslinked alginate nanoparticles were recorded at different pH values, namely 4.5, 7.4, and 10, using the dynamic light scattering (DLS) technique.

### 3.9. Statistical Analysis

All data were analysed using two-way ANOVA, multiple comparisons, and compare column means (main column effect) using GraphPad Prism software version 9.4.0 and are included in the Supplementary File (Tables S1–S12). All measurements were conducted in triplicates (*n* = 3), and the mean values of triplicates were calculated.

## 4. Results and Discussion

At pH higher than 3.5, i.e., greater than the pKa of the carboxyl groups (–COO⁻) on alginate residues, the alginate chains become ionised due to the deprotonation of their carboxyl groups [43]. This allows for crosslinking with divalent cations like calcium. On the other hand, at low pH (below ~3.5), these carboxyl groups become protonated to form –COOH, which is the unionised form of alginate carboxylic acid groups. This reduces the ability of alginate chains to bind with calcium ions and prevents them from forming the entanglement required for gel, microparticle, and nanoparticle formation. However, alginate could be crosslinked through hydrogen bonding between protonated carboxyl groups [44]. Also, at low pH, alginate can undergo physical gelation via aggregation of the polymer chains due to hydrophobic interactions. Therefore, in this study, microfluidics was used to investigate the possibility of producing alginate nanoparticles based on the acidification of an alginate solution at pH below alginate pKa, i.e., 3.5. As a proof of concept, alginate nanoparticles were prepared using alginate of two different molecular weights, including low molecular weight and high molecular weight alginate, which were denoted as low and high alginate, respectively, and at two different concentrations, which were 0.2 (*w*/*w*%) and 0.4 (*w*/*w*%).

### 4.1. Characterisation of Acid-Gelled Blank Alginate Nanoparticles

**Figure 10:**
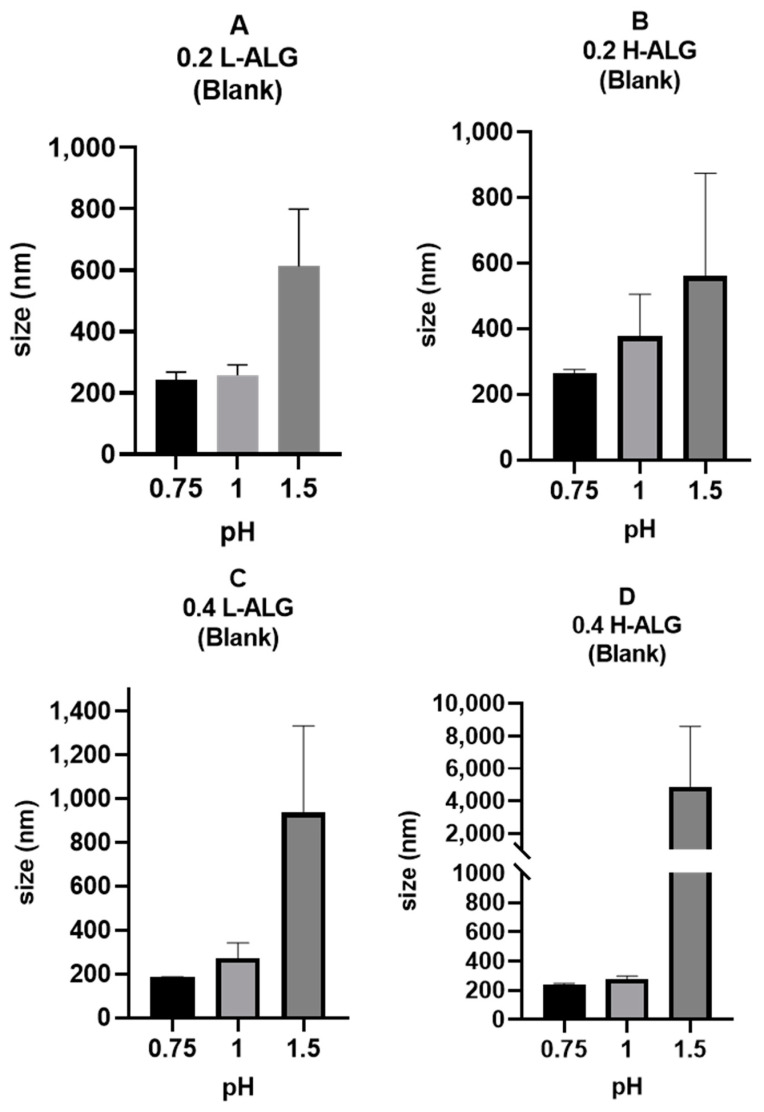
Hydrodynamic diameters of acid-gelled alginate nanoparticles unloaded with drug (blank) using hydrochloric acid as a crosslinking agent at different molar concentrations with different pH values: 0.75, 1, and 1.5, respectively. (**A**) Acid-gelled alginate nanoparticles prepared with 0.2% (*w*/*w*) low-viscosity sodium alginate. (**B**) Acid-gelled alginate nanoparticles prepared with 0.2% (*w*/*w*) high-viscosity sodium alginate. (**C**) Acid-gelled alginate nanoparticles prepared with 0.4% (*w*/*w*) low-viscosity sodium alginate at TL. (**D**) Acid-gelled alginate nanoparticles prepared with 0.4% (*w*/*w*) high-viscosity sodium alginate.

Figure 10 demonstrates the mean hydrodynamic diameter (MHD) of blank alginate nanoparticles before loading the drug into the nanoparticles. It is obvious that the pH of the gelling solution (HCl solution) is the key factor that affects the particle size, where the mean size of the produced alginate nanoparticles increased as the pH value was raised from 0.75 to 1.5. Specifically, at pH 1.5, a significant increase in the particle size was observed, even if this pH value is 2 pH units below the pKa of alginate carboxylic acid groups, meaning that they must be 99% unionised. This can be explained through the fact that not all carboxyl on alginate have the same pKa, and, therefore, some of them could still be protonated at pH 1.5 (i.e., ionised). The ionisation of carboxylic acid groups could cause an electrostatic repulsion that leads to more hydrated particles with larger hydrodynamic diameters in comparison with alginate nanoparticles prepared by acidification at lower pH values such as 0.75, where alginate carboxylic acid groups are completely unionised, excluding any possible electrostatic repulsive forces; this has also been described previously [45].

**Figure 11:**
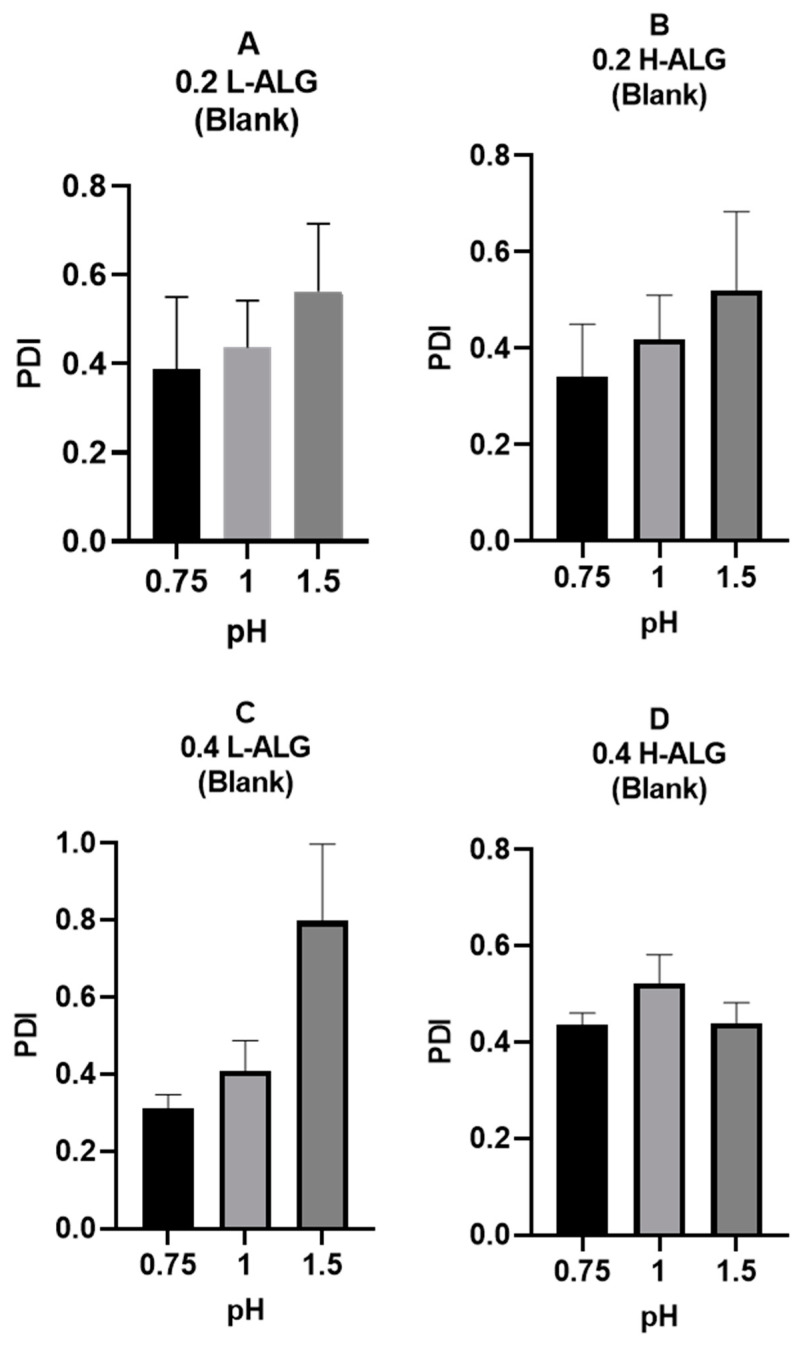
Polydispersity index (PDI) of acid-gelled alginate nanoparticles unloaded with drug (blank) using hydrochloric acid as a crosslinking agent at different molar concentrations with different pH values: 0.75, 1, and 1.5, respectively. (**A**) Acid-gelled alginate nanoparticles prepared with 0.2% (*w*/*w*) low-viscosity sodium alginate. (**B**) Acid-gelled alginate nanoparticles prepared with 0.2% (*w*/*w*) high-viscosity sodium alginate. (**C**) Acid-gelled alginate nanoparticles prepared with 0.4% (*w*/*w*) low-viscosity sodium alginate at TL. (**D**) Acid-gelled alginate nanoparticles prepared with 0.4% (*w*/*w*) high-viscosity sodium alginate.

Similarly, the polydispersity index (PDI) values of the produced alginate nanoparticles rose as the pH of the acidification/gelling medium increased from 0.75 to 1.5, indicating poor particle characteristics at higher pH values, as shown in Figure 11.

The effect of the viscosity of alginate on the physical properties of alginate nanoparticles can be grasped most clearly at high molecular weight and high concentration of alginate, where alginate nanoparticles displayed relatively greater sizes and polydispersity index values as the concentration of alginate and its molecular weight increased.

Regarding the variation in the molecular weight of alginate and its influence on the resultant nanoparticles, viscosity would be a prominent factor that is linked to alginate molecular weight that could affect the formation of alginate nanoparticles; the viscosity of alginate solution would increase as the molecular weight of alginate increases at specific concentrations [45]. Figure 10 also shows that the MHD of alginate nanoparticles increased as the molecular weight of alginate used for nanoparticle production increased at specific alginate concentrations, and the effect was more prominent at both pH 1.5 and a high alginate concentration of 0.4% (*w*/*v*), i.e., Figure 10C vs. Figure 10D. This could be attributed to the fact that the viscosity of alginate solution commonly increases when the molecular weight increases.

Viscosity is a critical parameter in the production of alginate nanoparticles using microfluidic systems, as mixing between the alginate solution and the acidification/gelling medium occurs primarily by diffusion into the microfluidic channel [46]. The high viscosity of the fluid (high molecular weight alginate solution) reduces the diffusion rate, leading to lower mixing capacity and rate [47] and increased potential for the formation of alginate nanoparticles that have larger diameters, as well as higher polydispersity index values (PDIs), i.e., less uniform particles. In contrast, lower-viscosity fluid (low molecular weight alginate) could promote rapid and uniform mixing; consequently, this would result in alginate nanoparticles with lower hydrodynamic diameters and narrower size distributions.

Moreover, a high-viscosity solution (high molecular weight alginate) could delay the nucleation of the produced nanoparticles by hindering molecular diffusion [48,49]. This delay can lead to the formation of nanoparticles with larger diameters or particles with more variation in diameters (higher polydispersity). On the other hand, a low-viscosity solution (low molecular weight alginate) promotes faster nucleation and more uniform particle growth, resulting in alginate nanoparticles with smaller diameters and lower PDIs compared to the alginate nanoparticles prepared using high molecular weight alginate. However, as illustrated in Figure 11, the PDI values of acid-gelled blank alginate nanoparticles were not greatly influenced by alginate molecular weight, in contrast with alginate nanoparticles’ MHD, which was more affected by alginate molecular weight. It also can be noted that at a lower pH of 0.75, all acid-gelled blank alginate nanoparticle values, regardless of alginate molecular weight, displayed a relatively lower PDI than alginate nanoparticles prepared at higher pH values of the gelling/acidification medium.

**Figure 12:**
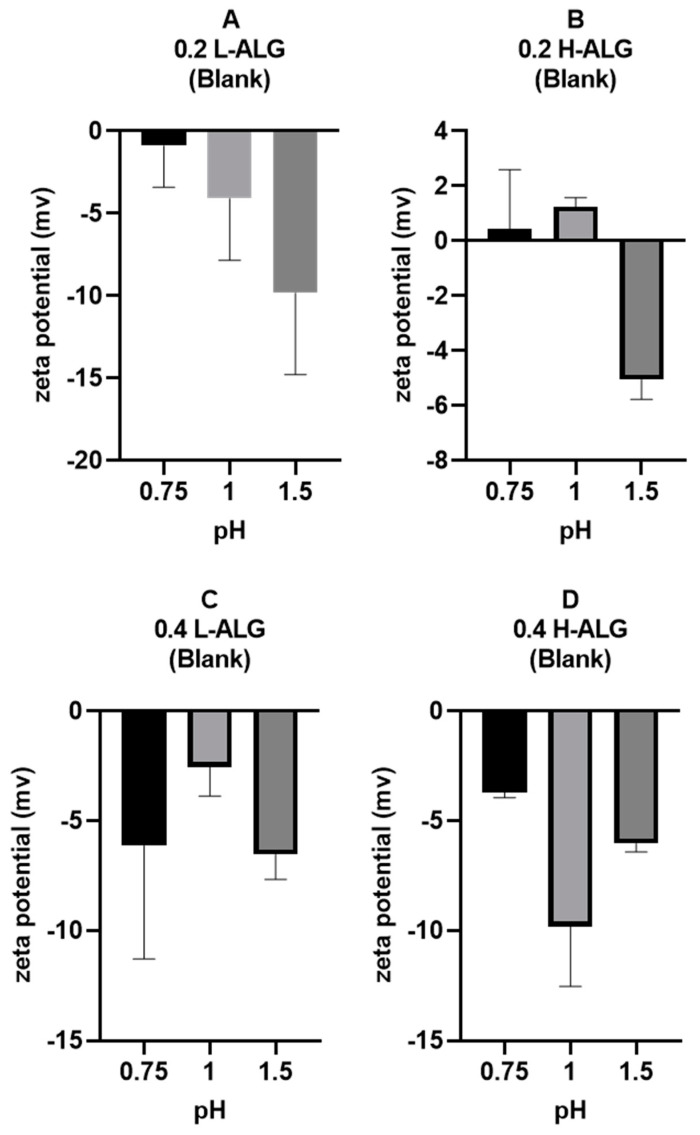
Zeta potential values of acid-gelled alginate nanoparticles unloaded with drug (blank) using hydrochloric acid as a crosslinking agent at different molar concentrations with different pH values: 0.75, 1, and 1.5, respectively. (**A**) Acid-gelled alginate nanoparticles prepared with 0.2% (*w*/*w*) low-viscosity sodium alginate. (**B**) Acid-gelled alginate nanoparticles prepared with 0.2% (*w*/*w*) high-viscosity sodium alginate. (**C**) Acid-gelled alginate nanoparticles prepared with 0.4% (*w*/*w*) low-viscosity sodium alginate at TL. (**D**) Acid-gelled alginate nanoparticles prepared with 0.4% (*w*/*w*) high-viscosity sodium alginate.

Furthermore, zeta potential measurements of alginate nanoparticles are illustrated in Figure 12. It could be observed that the zeta potential values at pH 0.75 and 1 are about zero (neutral), thus suggesting that the majority of –COOH groups are protonated (unionised). However, at pH 1.5, the zeta potential values were relatively negative, indicating the ionisation of some carboxylic acid at the surfaces of particles. This is more obvious at a low concentration of alginate (0.2% *w*/*w*), with some slightly positive values likely due to the absorption of hydronium ions onto the surfaces of the nanoparticles. At a high concentration of alginate, it seems that the zeta potential values were almost indifferent.

### 4.2. Characterisation of Ca^+2^-Crosslinked Blank Alginate Nanoparticles

**Figure 13:**
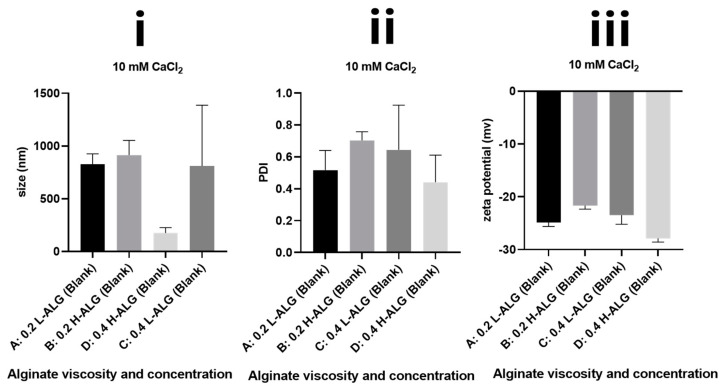
Hydrodynamic diameters (**i**) polydispersity index/PDI and (**ii**) zeta potential values of (**iii**) Ca^+2^-crosslinked alginate nanoparticles unloaded with 5-FU (blank). A: Ca^+2^-crosslinked gelled alginate nanoparticles prepared with 0.2% (*w*/*w*) low-viscosity sodium alginate. B: Ca^+2^-crosslinked alginate nanoparticles prepared with 0.2% (*w*/*w*) high-viscosity sodium alginate. C: Ca^+2^-crosslinked alginate nanoparticles prepared with 0.4% (*w*/*w*) low-viscosity sodium alginate. D: Ca^+2^-crosslinked alginate nanoparticles prepared with 0.4% (*w*/*w*) high-viscosity sodium alginate.

In comparison with alginate nanoparticles prepared by acid gelation, calcium-crosslinked alginate nanoparticles demonstrated relatively higher mean hydrodynamic diameters and greater polydispersity index values, apart from Ca^+2^-crosslinked alginate nanoparticles prepared at 0.4% (*w*/*w*) using high molecular weight alginate (Figure 13). However, even these calcium-crosslinked nanoparticles showed a higher polydispersity index greater than 0.4, meaning that alginate nanoparticles produced by this method are not optimum for drug delivery applications. Most importantly, the zeta potential values of all prepared Ca^+2^-crosslinked alginate nanoparticles were negative due to the presence of carboxylate groups exposed on the surfaces of alginate nanoparticles. Albeit the zeta potential absolute values are relatively high (greater than 20), which could increase the physical stability of the nanoparticles by virtue of their electrostatic repulsive forces. The produced alginate nanoparticles showed higher particle size and PDI values in comparison with blank nanoparticles prepared by acid gelation. This indicates that the poor characteristics of the nanoparticles are not due to the physical instability and subsequent particle aggregation but are rather owing to the improper crosslinking and subsequent irregular growth of the alginate nanoparticles produced by divalent cation (Ca^+2^) crosslinking. This implies that the acid gelation method is superior compared to calcium gelation as a robust technique for the production of alginate nanoparticles.

### 4.3. Characterisation of 5-FU-Loaded Acid-Gelled Alginate Nanoparticles

The encapsulation/association of the drug 5-fluorouracil (5-FU) can significantly affect the size of alginate nanoparticles due to several factors related to drug properties and the interaction between the drug and alginate matrix during the encapsulation/association process.

The ionisation state of the drug could also affect the characteristics of the drug-loaded nanoparticles. The alginate chain possesses negatively charged carboxylate groups, which can interact with the loaded drugs based on their ionisation states/charges. Positively charged drugs can form strong electrostatic interactions with the alginate matrix, potentially affecting the particle size [50]. These interactions could compact the nanoparticle by drawing the alginate molecules closer together. On the other hand, negatively charged drugs could form repulsive forces between alginate chains, thus expanding the produced nanoparticles by influencing the structure of the nanoparticle matrix [51].

5-fluorouracil (5-FU) is a water-soluble drug possessing two pKa values, which are 7.76 and 8.23, resulting in the ionisation of two acidic hydrogens (deprotonation) in its structure when the pH of the medium is higher than those pKa values [52]. However, during the preparation of alginate nanoparticles using the acidification method, the processing medium was acidic, with a pH much lower than the stated pKa values of 5-FU, rendering the drug molecules essentially protonated rather than negatively charged. This means the ionisation state of 5-FU did not influence the diameters of the proton/acid-gelled alginate nanoparticles by virtue of drug–polymer electrostatic interaction.

On the other hand, hydrophilic drugs like 5-FU typically integrate well into the aqueous phase of the alginate gel, resulting in uniform encapsulation [53]. Depending on the concentration of the added drug, they may increase particle size by expanding the matrix, but they are less likely to cause significant aggregation or irregular growth [54].

**Figure 14:**
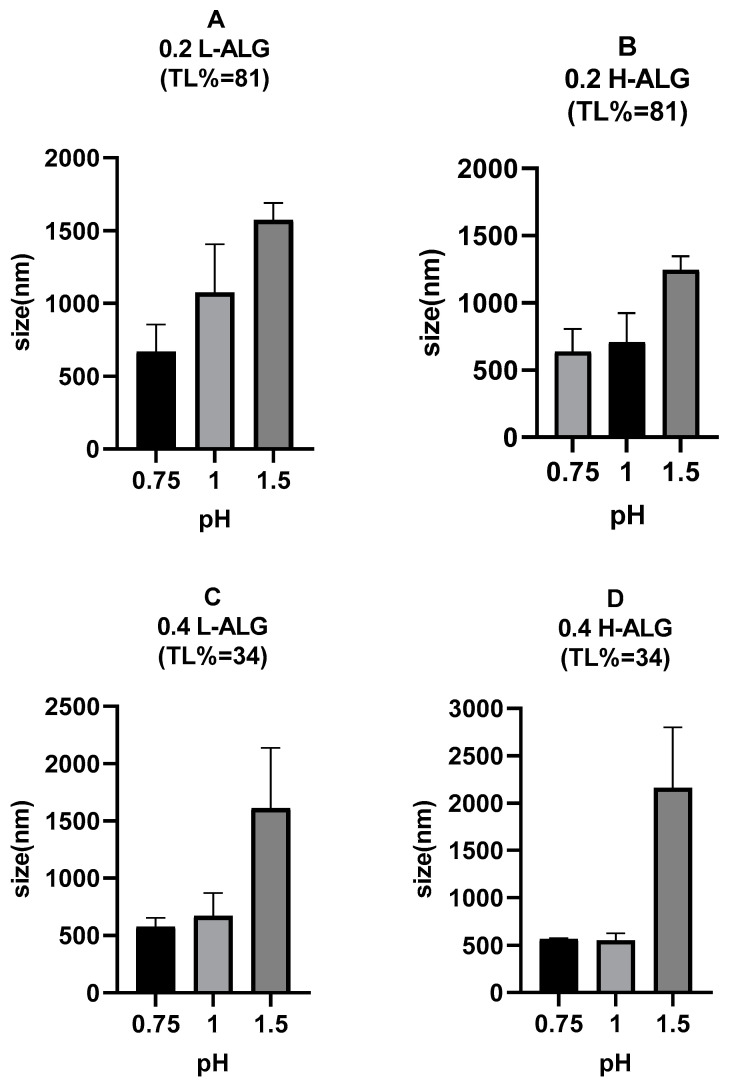
Hydrodynamic diameters of acid-gelled alginate nanoparticles loaded with 5-FU at different theoretical loadings (TLs), 34% and 81%, using hydrochloric acid as a crosslinking agent at different molar concentrations with different pH values: 0.75, 1, and 1.5, respectively. (**A**) Acid-gelled alginate nanoparticles prepared with 0.2% (*w*/*w*) low-viscosity sodium alginate at TL of 81%. (**B**) Acid-gelled alginate nanoparticles prepared with 0.2% (*w*/*w*) high-viscosity sodium alginate at TL of 81%. (**C**) Acid-gelled alginate nanoparticles prepared with 0.4% (*w*/*w*) low-viscosity sodium alginate at TL of 34%. (**D**) Acid-gelled alginate nanoparticles prepared with 0.4% (*w*/*w*) high-viscosity sodium alginate at TL of 34%.

Figure 14 shows a dramatic increase in the mean hydrodynamic diameters of the 5-FU-loaded alginate nanoparticles compared to blank (drug-unloaded) alginate nanoparticles prepared by the acidification technique, supporting the general theory that assumes the drug encapsulation process could expand the sizes of the particles.

The figure also demonstrates that the mean hydrodynamic diameters of 5-FU-loaded nanoparticles rose while the theoretical loading (TL) of the drug increased from 34% at a high alginate concentration of 4% (Figure 14C,D) to 81% at a low alginate concentration of 0.2% (*w*/*v*) (Figure 14A,B). This means that when the concentration of the drug within the nanoparticle increased, there was an increase in the nanoparticle size. This could be explained by the fact that more drug molecules need to be accommodated within the alginate matrix, which can lead to a looser structure or more extensive crosslinking, both of which contribute to larger particle size [55]. However, this effect was only observed for the alginate nanoparticles produced at pH values of 0.75 and 1 but not for alginate nanoparticles produced at pH 1.5. This is thought to be due to the effect of alginate concentration, which would become more predominant at pH 1.5 with a more extended polymeric matrix, resulting in alginate particles with larger diameters even though theoretical loading is lower, i.e., the added amount of drug was lower (0.05% (*w*/*v*) for 34% TL vs. 0.2% (*w*/*v*) for 81% TL).

Moreover, the viscosity of the drug-containing solution can also affect the mean hydrodynamic diameters of 5-FU-loaded alginate nanoparticles [56]. A higher viscosity of the HCl solution used for the proton gelation of alginate nanoparticles is speculated due to the dissolved drug in comparison with drug-free HCl solution. This is thought to slow down the mixing and diffusion of the fluids during nanoparticle formation, potentially leading to particles with larger diameters due to slower nucleation and growth in comparison with blank alginate nanoparticles.

Additionally, a similar trend was observed for 5-FU-loaded alginate nanoparticles regarding the pH of the HCl/gelling solution, where the mean hydrodynamic diameters also increased as the pH value increased from 0.75 to 1.5. The effect of alginate viscosity linked to the molecular weight of alginate on the mean hydrodynamic diameters was also prominent in 5-FU-loaded alginate nanoparticles, and an incremental particle size was observed for 5-FU-loaded alginate nanoparticles when using high molecular weight alginate in comparison with low molecular weight grade.

**Figure 15:**
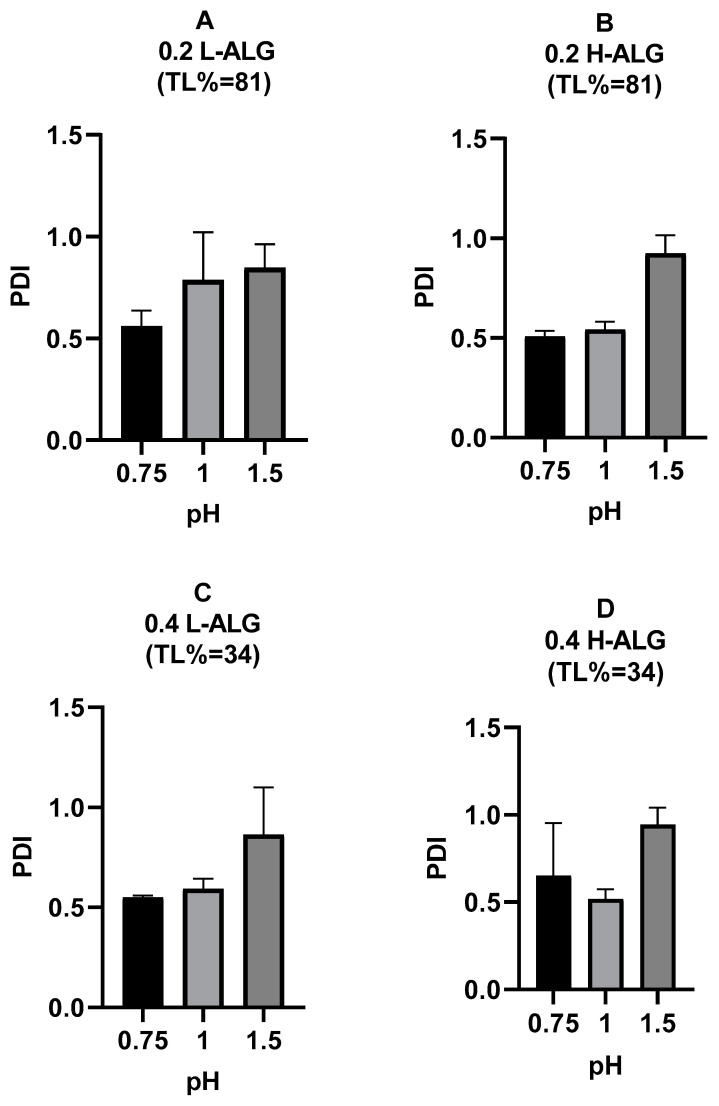
Polydispersity index (PDI) of acid-gelled alginate nanoparticles loaded with 5-FU at different theoretical loadings, 34% and 81%, using hydrochloric acid as a crosslinking agent at different molar concentrations with different pH values: 0.75, 1, and 1.5, respectively. (**A**) Acid-gelled alginate nanoparticles prepared with 0.2% (*w*/*w*) low-viscosity sodium alginate at TL of 81%. (**B**) Acid-gelled alginate nanoparticles prepared with 0.2% (*w*/*w*) high-viscosity sodium alginate at TL of 81%. (**C**) Acid-gelled alginate nanoparticles prepared with 0.4% (*w*/*w*) low-viscosity sodium alginate at TL of 34%. (**D**) Acid-gelled alginate nanoparticles prepared with 0.4% (*w*/*w*) high-viscosity sodium alginate at TL of 34%.

Figure 15 also shows the polydispersity index values of 5-FU-loaded acid-gelled alginate nanoparticles; they displayed PDI values of 0.5 or more regardless of alginate molecular weight or alginate concentration. However, for 5-FU-loaded acid-gelled alginate prepared at low pH values of 0.75, the nanoparticles demonstrated lower PDI values compared with alginate nanoparticles prepared at higher pH values, i.e., 1.5.

**Figure 16:**
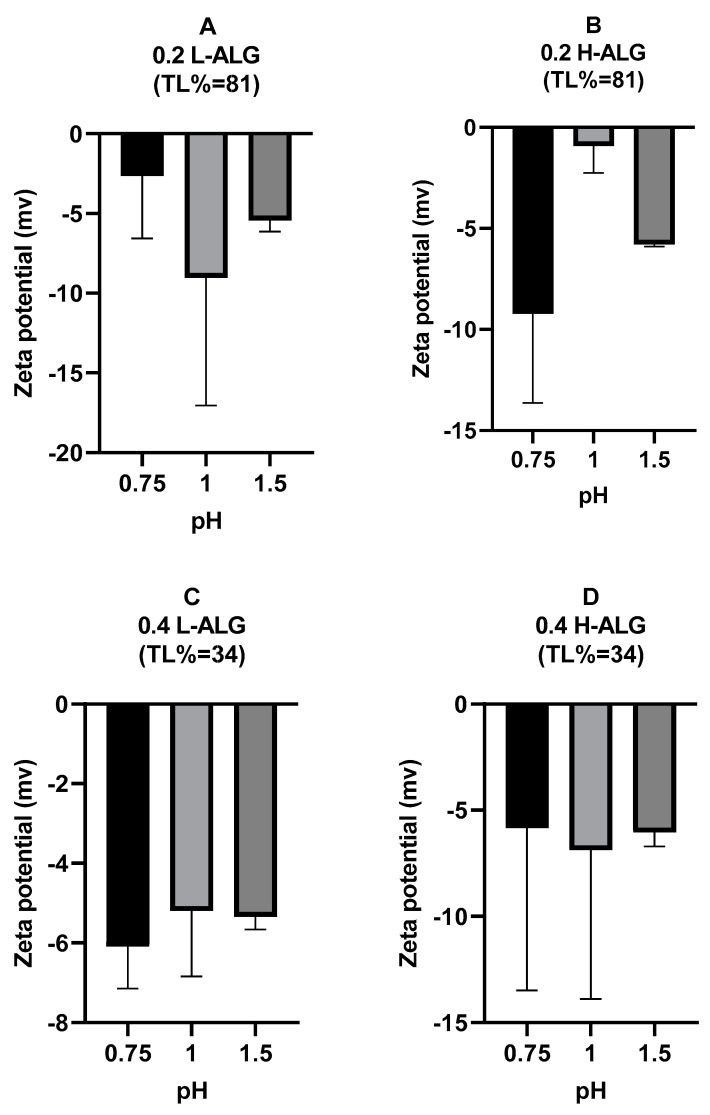
Zeta potential of acid-gelled alginate nanoparticles loaded with 5-FU at different theoretical loadings (TLs), 34% and 81%, using hydrochloric acid as a crosslinking agent at different molar concentrations with different pH values: 0.75, 1, and 1.5, respectively. (**A**) Acid-gelled alginate nanoparticles prepared with 0.2% (*w*/*w*) low-viscosity sodium alginate at TL of 81%. (**B**) Acid-gelled alginate nanoparticles prepared with 0.2% (*w*/*w*) high-viscosity sodium alginate at TL of 81%. (**C**) Acid-gelled alginate nanoparticles prepared with 0.4% (*w*/*w*) low-viscosity sodium alginate at TL of 34%. (**D**) Acid-gelled alginate nanoparticles prepared with 0.4% (*w*/*w*) high-viscosity sodium alginate at TL of 34%.

Regarding the zeta potential values of the 5-FU-loaded alginate nanoparticles, the zeta potential was not greatly affected by 5-FU loading, and all 5-FU-loaded alginate nanoparticle formulations possessed a slightly negative charge (overall zeta potential values were less than −10), as illustrated in Figure 16. This could be attributed to the fact that the 5-FU is essentially unionised at all pH values of the acidification/gelling medium, i.e., pH 0.75, 1, and 1.5. Only ionised drugs would influence the zeta potential values of the drug-loaded nanoparticles, specifically if they become predominantly adsorbed onto the surfaces of the produced nanoparticles.

### 4.4. Characterisation of 5-FU-Loaded Ca^+2^-Crosslinked Alginate Nanoparticles

**Figure 17:**
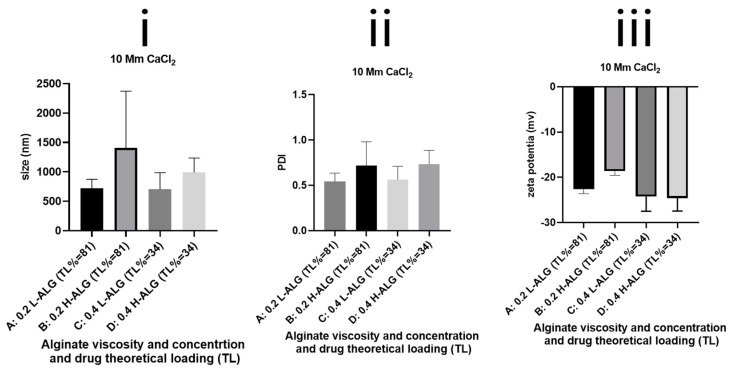
Hydrodynamic diameters (**i**), polydispersity index/PDI (**ii**), and zeta potential values (**iii**) of Ca^+2^-crosslinked alginate nanoparticles loaded with 5-FU at different theoretical loadings, 34% and 81%, using calcium chloride as a crosslinking agent at a molar concentration of 10 mM. A: Ca^+2^-crosslinked alginate nanoparticles prepared with 0.2% (*w*/*w*) low-viscosity sodium alginate at 81%. B: Ca^+2^-crosslinked alginate nanoparticles prepared with 0.2% (*w*/*w*) high-viscosity sodium alginate at 81%. C: Ca^+2^-crosslinked alginate nanoparticles prepared with 0.4% (*w*/*w*) low-viscosity sodium alginate at 34%. D: Ca^+2^-crosslinked alginate nanoparticles prepared with 0.4% (*w*/*w*) high-viscosity sodium alginate at 34%.

**Figure 18:**
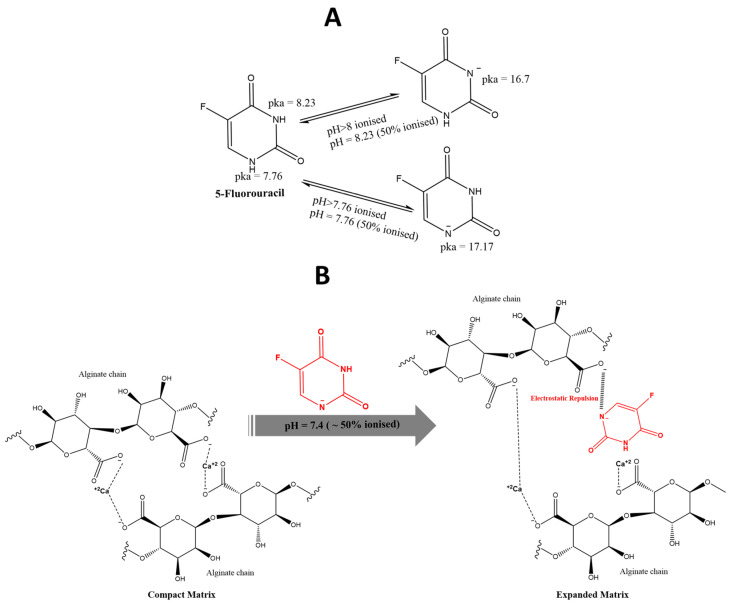
Schematic diagram illustrating the proposed mechanistic explanation for the effect of 5-FU (red) incorporation on the matrix of Ca^+2^-crosslinked alginate nanoparticles. (**A**) The ionisation states of 5-FU possessing 2 pKa values of its acidic groups. (**B**) The compact structure of the alginate matrix of Ca^+2^-crosslinked alginate nanoparticles in comparison with the expanded structure of the alginate matrix of the same nanoparticles due to incorporating 5-FU (red) into the nanoparticles as a result of the electrostatic repulsive forces between negatively charged 5-FU and negatively charged alginate carboxylate groups.

Some drugs may interfere with the crosslinking process, either by competing with calcium ions or by forming complexes with the alginate itself [57]. This could lead to a less compact structure, increasing nanoparticle size. In the case of 5-FU-loaded calcium-crosslinked nanoparticles, the obtained alginate particles demonstrated larger diameters than the blank calcium-crosslinked alginate nanoparticles, as demonstrated in Figure 17. This could be explained by the fact that the presence of 5-FU could affect the efficiency of the crosslinking between the negatively charged alginate chains and the divalent cation, i.e., calcium. In the case of 5-FU, as the pH of the processing medium was 7.4, this could have rendered 5-FU partially ionised, where it has a pKa value of 7.6, and at this pH, the 5-FU could form negatively charged species (almost half-ionised), which, in turn, could greatly affect the crosslinking process between alginate chains and the divalent cation, i.e., Ca^+2^, by creating more electrostatic repulsive interactions with the alginate polymer chains. Thus, this would result in a less compact or more expanded nanostructure of the produced 5-FU alginate nanoparticles crosslinked with calcium; this proposed mechanistic explanation is illustrated in the schematic diagram in Figure 18. It was also observed that the molecular weight of alginate influenced the diameters of the produced nanoparticles, which could be attributed to a more extended structure brought by longer alginate chains (i.e., high MW); a similar finding was reported previously [58]. Figure 17 also shows the polydispersity index values of Ca^+2^-crosslinked alginate nanoparticles loaded with 5-FU, and these values were greater than 0.5 for all formulations, indicating poor uniformity of the particle diameters produced by Ca^+2^ crosslinking after addition of the drug. This also reinforces that calcium crosslinking is not an efficient method for preparing 5-FU-loaded alginate nanoparticles compared to the acidification/proton gelation method in terms of nanoparticle characteristics.

With respect to the zeta potential values of 5-FU-loaded Ca^+2^-crosslinked nanoparticles, all formulations displayed negative zeta potential values greater than 25 (i.e., the absolute value), which further affirms that the larger particle diameters are not due to physical instability and aggregate formation but due to the imperfection of the crosslinking process disrupted by the added drug: 5-FU. It could also be concluded that the addition of the 5-FU drug to Ca^+2^-crosslinked alginate nanoparticles did not greatly affect the zeta potential values compared to blank Ca^+2^-crosslinked alginate nanoparticles, which could indicate that the drug was not adsorbed to the surface of the nanoparticles, especially suggesting that the drug was likely entrapped within the alginate matrix, disrupting the crosslinking process.

### 4.5. Encapsulation Efficiency of 5-FU Loaded into Alginate Nanoparticles

#### 4.5.1. Encapsulation Efficiency of 5-FU Loaded into Acid-Gelled Alginate Nanoparticles

**Figure 19:**
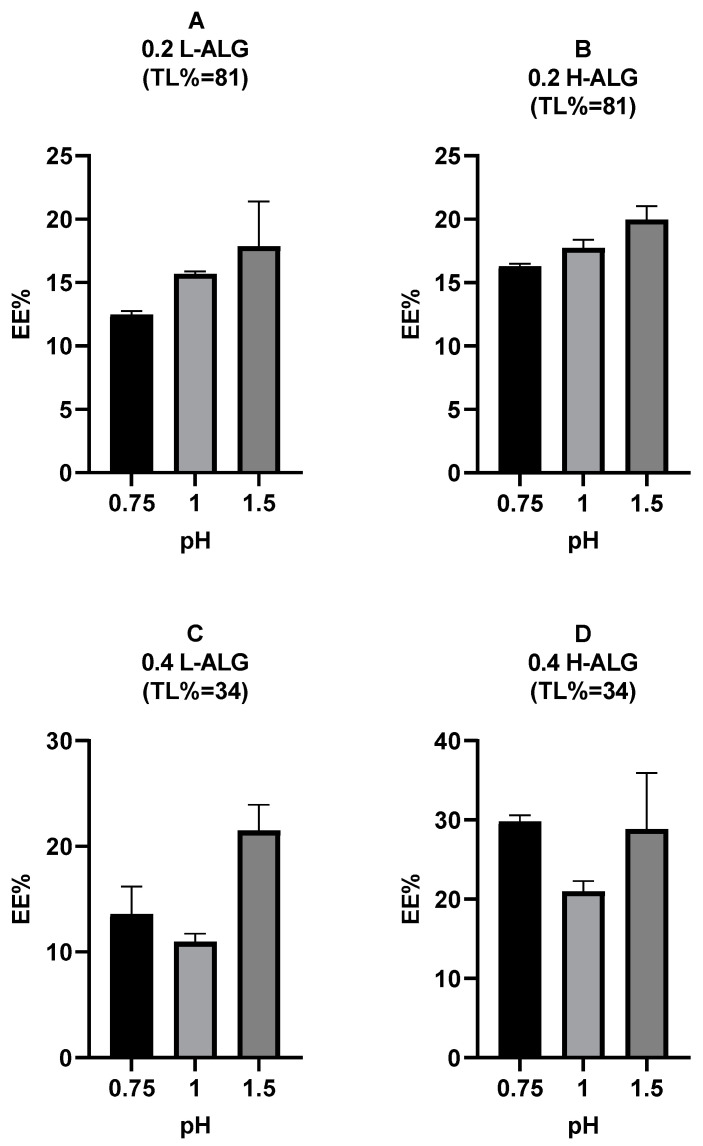
Encapsulation efficiency (EE%) of 5-FU loaded into acid-gelled alginate nanoparticles at different theoretical loadings (TLs) of 34% and 81%. (**A**) Acid-gelled alginate nanoparticles prepared with 0.2% (*w*/*w*) low-viscosity sodium alginate at TL of 81%. (**B**) Acid-gelled alginate nanoparticles prepared with 0.2% (*w*/*w*) high-viscosity sodium alginate at TL of 81%. (**C**) Acid-gelled alginate nanoparticles prepared with 0.4% (*w*/*w*) low-viscosity sodium alginate at TL of 34%. (**D**) Acid-gelled alginate nanoparticles prepared with 0.4% (*w*/*w*) high-viscosity sodium alginate at TL of 34%.

The encapsulation efficiency (EE%) of 5-FU loaded into alginate nanoparticles prepared by acid/proton gelation was calculated for each formulation, as illustrated in Figure 19. It was observed that at high theoretical loading (TL = 81%) and 0.2% (*w*/*v*) alginate polymer concentration, the EE% of the 5-FU into alginate nanoparticles was increased slightly when high molecular weight alginate (H-ALG) was used in comparison with low molecular weight alginate (L-ALG). A similar trend was seen when using 0.4% (*w*/*v*) and low theoretical loading (TL = 34%) with a higher increment in the EE% due to the increase in the alginate molecular weight relative to the high theoretical loading. It was also observed that there was an increase in the EE% when the pH of the acidification/gelling medium was raised at a high drug TL of 81% and a low alginate concentration of 0.2% (*w*/*v*), which is thought to be due to higher particle diameters that would accommodate more drug molecules. However, this trend was not observed at a low drug TL of 34% and a high alginate concentration of 0.4% (*w*/*v*). With low molecular weight alginate, increasing the theoretical loading from 34% to 81% TL (Figure 19A vs. Figure 19C) did not lead to any increase in the EE% of 5-FU into alginate nanoparticles. On the other hand, at high molecular weight alginate, there was no increase in the EE% of 5-FU into alginate nanoparticles when the theoretical loading increased from 34% to 81% TL (Figure 19B vs. Figure 19D), apart from 5-FU-loaded alginate nanoparticles prepared at pH 0.75, where the EE% was even reduced when the theoretical loading was high. This could be attributed to the low concentration of the alginate polymer (0.2%) at a high theoretical loading (81% TL) that had led to decreased drug entrapment due to reduced polymer capability to form a stable matrix that traps the drug. This phenomenon has been described in the literature previously [59]. This effect was not observed at pH 1 and 1.5, which is thought to be owing to the larger particle diameters produced at these pH values, which could lead to higher amounts of entrapped drug molecules in comparison with alginate nanoparticles prepared at pH 0.75. Overall, a high alginate concentration of 0.4% (*w*/*v*) and high molecular weight alginate at a theoretical loading (TL) of 34% (0.4 H-ALG (34%)), which was used to prepare acid-gelled alginate nanoparticles, could be considered the optimum formulation for 5-FU delivery.

#### 4.5.2. Encapsulation Efficiency of 5-FU Loaded into Ca^+2^-Crosslinked Alginate Nanoparticles

**Figure 20:**
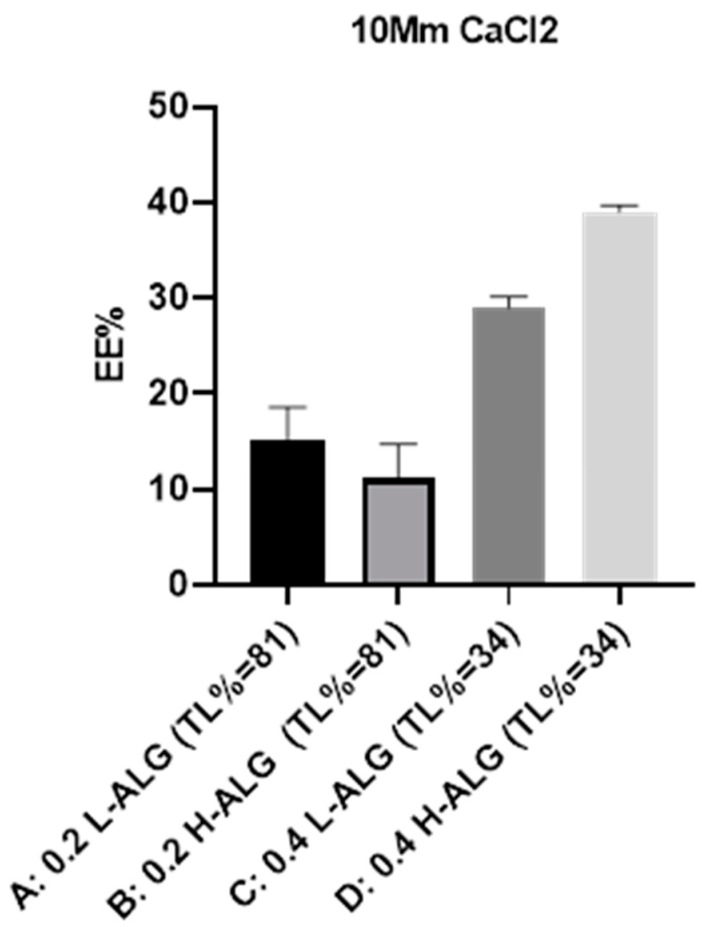
Encapsulation efficiency (EE%) of 5-FU-loaded Ca^+2^-crosslinked alginate nanoparticles at different theoretical loadings (TLs) of 34% and 81% using calcium chloride as a crosslinking agent at a molar concentration of 10 mM. (A) Ca^+2^-crosslinked alginate nanoparticles prepared with 0.2% (*w*/*w*) low-viscosity sodium alginate at TL of 81%. (B) Ca^+2^-crosslinked alginate nanoparticles prepared with 0.2% (*w*/*w*) high-viscosity sodium alginate at TL of 81%. (C) Ca^+2^-crosslinked alginate nanoparticles prepared with 0.4% (*w*/*w*) low-viscosity sodium alginate at TL of 34%. (D) Ca^+2^-crosslinked alginate nanoparticles prepared with 0.4% (*w*/*w*) high-viscosity sodium alginate at TL of 34%.

Figure 20 demonstrates the encapsulation efficiency (EE%) of 5-FU into alginate nanoparticles crosslinked with the divalent cation calcium. It was observed that at specific alginate molecular weights, i.e., Figure 20A vs. Figure 20C (low molecular weight alginate) and Figure 20B vs. Figure 20D (high molecular weight alginate), decreasing the theoretical loading of 5-FU led to an obvious increase in the encapsulation efficiency (EE%) of 5-FU into alginate nanoparticles. This could be explained by the fact that at lower drug theoretical loading (34% TL), the alginate concentration used to prepare 5-FU-loaded Ca^+2^-crosslinked alginate nanoparticles was higher (0.4% *w*/*v*) than that used to prepare the same alginate nanoparticles but at higher drug theoretical loading (81% TL) where the alginate concentration was 0.2% *w*/*v*. A higher alginate concentration could lead to a more stable alginate matrix with lower imperfections/pores, which could enhance the entrapment of drug molecules, which was described elsewhere [60]. Although the optimum formula for 5-FU loading is a high alginate concentration of 0.4% (*w*/*v*) and a high molecular weight alginate at a low theoretical loading of 34%, i.e., 0.4 H-ALG (TL = 34%), this formula displayed poor characteristics in terms of MHD (around 1000 nm) and PDI (greater than 0.5). Consequently, the optimum formula of Ca^+2^-crosslinked alginate nanoparticles is less favourable than the optimum formula of the newly developed acid-gelled alginate nanoparticles for the delivery of therapeutics, e.g., 5-FU.

### 4.6. Particle Integrity and Characteristics at Various pH Values (pH 4.5 and 10)

**Figure 21:**
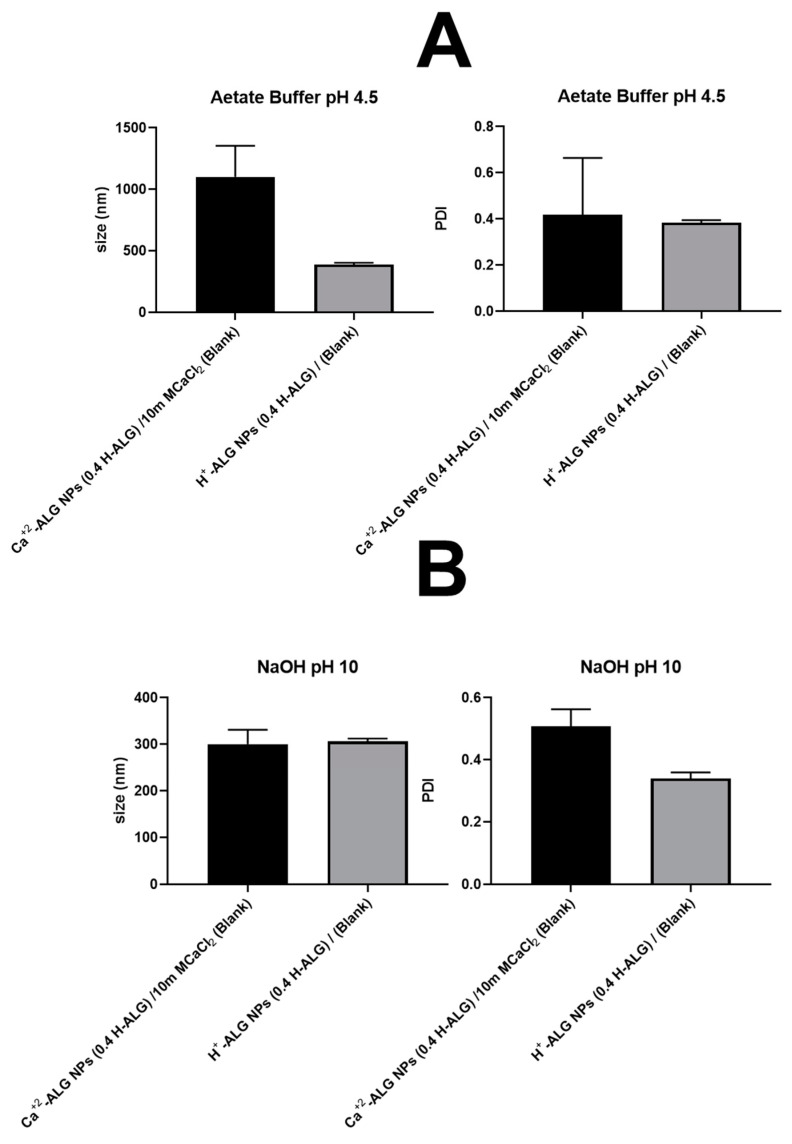
(**A**) Hydrodynamic diameter and polydispersity index (PDI) of acid-gelled alginate nanoparticles without 5-FU loading (blank) in comparison with Ca^+2^-crosslinked alginate nanoparticles without 5-FU loading (blank) prepared at high alginate viscosity and alginate concentration of 0.4 *w/v* % and placed at pH value of 4.5. (**B**) Hydrodynamic diameter and polydispersity index (PDI) of acid-gelled alginate nanoparticles without 5-FU loading (blank) in comparison with Ca^+2^-crosslinked alginate nanoparticles without 5-FU loading (blank) prepared at high alginate viscosity and alginate concentration of 0.4 *w/v* % and placed pH value of 10.

The integrity of acid-gelled blank alginate nanoparticles in terms of MHD and PDI was investigated at low and high pH values, specifically at pH 4.5 and 10, respectively, and compared with alginate nanoparticles prepared using ionotropic gelation with a divalent cation (Ca^+2^), as illustrated in Figure 21A,B. Acid-gelled blank alginate nanoparticles demonstrated nearly stable particle size/MHD and acceptable PDI when placed at either a low pH of 4.5 or an extremely high pH of 10 (Figure 11A vs. Figure 11B). This means that alginate nanoparticles did not lose their integrity due to high pH, and there is the possibility of alginate carboxylate group ionisation (deprotonation) within the matrix of the nanoparticles and the subsequent loss of hydrogen bonds and the formation of repulsive forces. This could be attributed to the high crosslinking density of acid-gelled alginate nanoparticle matrices, which is caused by the protonation of alginate carboxylate groups and the subsequent formation of stable H-bonds. A higher density of alginate chain crosslinking could restrict ion/proton mobility and alter the protonation state of the carboxyl groups on the alginate chains [61]. This can modify the pKa by affecting the ease with which protons dissociate from the carboxyl groups, thus reducing the ability of protons to dissociate from alginate carboxylic acid groups within the alginate nanoparticle matrix and retaining its integrity and stability by maintaining stable H-bonds between alginate polymeric chains [62].

On the other hand, when Ca^+2^-crosslinked blank alginate nanoparticles were placed at a high pH value of 10 (Figure 11B), alginate nanoparticles displayed a lower particle size/MHD. This could be attributed to the fact that at pH 10, which is more than 3 pH units above alginate carboxylate groups’ pKa (3.5), these groups that were attached to the surface of alginate nanoparticles became fully ionised, increasing the zeta potential and resulting in high electrostatic repulsive interactions between the adjacent alginate nanoparticles; this would prevent any possible aggregation and size enlargement. In contrast, at pH 4.5, i.e., an acidic environment (Figure 11A), alginate carboxylate groups become less ionised (more protonated), shifting the nanoparticle’s surface charge (zeta potential); accordingly, this reduces the repulsion between adjacent alginate nanoparticles, thus enhancing the formation of large aggregates with an MHD greater than 1000 nm due to van der Waals forces (hydrophobic interactions) [63]. Overall, acid-gelled alginate nanoparticles demonstrated superior particle integrity at different pH values, meaning that the proton gelation method produced alginate nanoparticles with stable alginate matrices that could withstand any change in the local environment, such as the pH, without changing the structural compactness of the alginate nanoparticles’ matrices.

## 5. Conclusions and Future Perspectives

This study has demonstrated that alginate nanoparticles could be synthesised using a novel green method relying on the acidification of alginate chains and the formation of stable H-bonds between the polymeric chains due to the protonation of alginate carboxylic acid groups in order to produce acid-gelled alginate nanoparticles. This invented technology is not only devoid of solvent usage but also eliminates the need for using multivalent cations, e.g., Ca^+2^, by relying on a lab-on-a-chip technique with hydrodynamic flow focused mixing of the fluids in order to produce less dispersed and more homogeneous nanoparticles. This novel technology provides new advantages over the conventional ionotropic gelation method that relies on using multivalent cations to crosslink alginate chains during nanoparticle formation, which could eventually lead to cation leakage from the nanoparticle’s matrix during the ion exchange process, thus disrupting the integrity of the nanoparticles and leading to matrix degradation and dissolution and thus impacting the nanoparticles‘ capability of holding drug cargo. Additionally, this green technology is highly compliant with the sustainable development goals (SDGs) set by the United Nations (UN), which stress reducing the consumption of organic solvents that could have a devastating influence on the ecosystem. The study demonstrated that acid-gelled nanoparticles displayed different particle characteristics in terms of MHD, PDI, and zeta potential by varying several factors, including the pH of the acidification/gelling medium and the concentration and molecular weight of alginate. Overall, acid-gelled blank alginate nanoparticles showed an MHD of circa 200 nm and an acceptable PDI of less than 0.4 at a low pH value, i.e., 0.75, of the acidification/gelling medium irrespective of alginate molecular weight, which could be attributed to the formation of more compact alginate matrices of alginate nanoparticles produced by the proton gelation method at the lowest pH value. On the other hand, acid-gelled blank alginate nanoparticles prepared at pH 0.75 and a higher alginate concentration (0.4%) displayed lower values of MHD and PDI in comparison with the corresponding alginate nanoparticles prepared at a low pH of 0.75 and a low alginate concentration, which is likely due to the formation of more stable and packed alginate matrices of the nanoparticles. Alginate nanoparticles demonstrated low zeta potential values, which were nearly zero at pH 0.75, due to the protonation of the carboxylate groups at the nanoparticles’ surfaces. In contrast, Ca^+2^-crosslinked blank alginate nanoparticles demonstrated greater MHD and PDI values in comparison with alginate nanoparticles produced by the acid-gelation method, although the zeta potential values of the produced nanoparticles were relatively high (greater than 20) and negative due to the deprotonation of carboxylic acid groups attached to the nanoparticles’ surfaces. This supports that the increase in the particle diameters is not due to particle aggregation and weak repulsive interactions between the particles but rather due to the loose structure of alginate matrices brought by unstable polymeric chain crosslinking by calcium. The designed system was successfully employed for 5-fluorouracil (5-FU) loading; however, the resultant 5-FU-loaded alginate nanoparticles produced by acid gelation displayed relatively higher MHD and PDI values compared with the corresponding blank alginate nanoparticles prepared using the same method. This could be ascribed to the increasing volume of alginate nanoparticles to accommodate more drug molecules with the nanoparticles’ matrices. A similar influence of factors such as pH, alginate concentration, and alginate molecular weight (viscosity) on the MHD and PDI was observed for 5-FU-loaded nanoparticles prepared by acid gelation. The integrity of alginate nanoparticles produced by the invented proton gelation method was investigated, and the findings were reinforced that the crosslinking density of alginate chains due to acid/proton gelation was stronger than crosslinking caused by the divalent calcium cation (Ca^+2^). This was clearly supported by the mean hydrodynamic diameters of acid-gelled nanoparticles, which remained nearly the same at various pH values, while the mean hydrodynamic diameters of Ca^+2^-crosslinked alginate nanoparticles increased at low pH values, indicating the formation of more stable alginate matrices prepared by the proton gelation method. Acid-gelled alginate nanoparticles demonstrated superior properties over Ca^+2^-crosslinked alginate nanoparticles in terms of MHD, PDI, drug encapsulation efficiency (EE%), and particle integrity at various pH values, i.e., low pH (4.5) and high pH (10). Accordingly, alginate nanoparticles produced by the invented green technology by exploiting the protons to induce alginate chains to crosslink through H-bonds could be further harnessed to produce drug-loaded nanoparticles for the delivery of therapeutics. In summation, proton/acid gelation used for the preparation of alginate nanoparticles could be considered a universal method for the synthesis of hydrophilic nanoparticles made of acidic polymers with the capability of entrapping anticancer drugs such as 5-FU with zero impact on the ecosystem.

## Figures and Tables

**Table 1:** Comparison of different nanoparticle synthesis methods. This table compares ionotropic gelation, acid gelation, and the emulsion method using an organic solvent, highlighting their principles, advantages, and limitations. The comparison emphasises the sustainability and biocompatibility of acid gelation as an eco-friendly alternative for drug delivery applications.

Method	Principle	Advantages	Limitations
Ionotropic Gelation	Crosslinking with divalent cations (e.g., Ca^+2^)	Mild conditions, high encapsulation efficiency, widely used	Requires external crosslinkers, possible batch-to-batch variability, ion leaching may disrupt nanoparticle integrity
Acid Gelation	Proton-driven gelation using acid	Green and sustainable, avoids toxic crosslinkers, simple process, devoid of cation leakage and nanoparticle’s matrix perpetration	Limited research, optimization needed for stability
Emulsion Method (Organic Solvent)	Formation of nanoparticles through emulsification in an organic solvent, followed by solvent evaporation	High drug loading, tunable particle size	Uses toxic solvents, requires multiple processing steps, potential residual solvent concerns

## Data Availability

The data presented in this study are available on request from the corresponding author.

## Supplementary Materials

The following supporting information can be downloaded at: https://www.mdpi.com/article/10.3390/pharmaceutics17040438/s1, Table S1: Hydrodynamic diameters of acid-gelled alginate nanoparticles unloaded with drug (blank) using hydrochloric acid as a crosslinking agent at different molar concentrations with different pH values: 0.75, 1, and 1.5, respectively; Table S2: Polydispersity index (PDI) of acid-gelled alginate nanoparticles unloaded with drug (blank) using hydrochloric acid as a crosslinking agent at different molar concentrations with different pH values: 0.75, 1, and 1.5, respectively; Table S3: Zeta potential values of acid-gelled alginate nanoparticles unloaded with drug (blank) using hydrochloric acid as a crosslinking agent at different molar concentrations with different pH values: 0.75, 1, and 1.5, respectively; Table S4: Hydrodynamic diameters, polydispersity index/PDI, and zeta potential values of Ca^+2^-crosslinked alginate nanoparticles unloaded with 5-FU (blank); Table S5: Hydrodynamic diameters of acid-gelled alginate nanoparticles loaded with 5-FU at different theoretical loadings (TLs), 34% and 81%, using hydrochloric acid as a crosslinking agent at different molar concentrations with different pH values: 0.75, 1, and 1.5, respectively; Table S6: Polydispersity index (PDI) of acid-gelled alginate nanoparticles loaded with 5-FU at different theoretical loadings, 34% and 81%, using hydrochloric acid as a crosslinking agent at different molar concentrations with different pH values: 0.75, 1, and 1.5, respectively; Table S7: Zeta potential of acid-gelled alginate nanoparticles loaded with 5-FU at different theoretical loadings (TLs), 34% and 81%, using hydrochloric acid as a crosslinking agent at different molar concentrations with different pH values: 0.75, 1, and 1.5, respectively; Table S8: Hydrodynamic diameters, polydispersity index/PDI, and zeta potential values of Ca^+2^-crosslinked alginate nanoparticles loaded with 5-FU at different theoretical loadings, 34% and 81%, using calcium chloride as a crosslinking agent at a molar concentration of 10 mM; Table S9: Encapsulation efficiency (EE%) of 5-FU loaded into acid-gelled alginate nanoparticles at different theoretical loadings (TLs) of 34% and 81%; Table S10: Encapsulation efficiency (EE%) of 5-FU-loaded Ca^+2^-crosslinked alginate nanoparticles at different theoretical loadings (TLs) of 34% and 81% using calcium chloride as a crosslinking agent at a molar concentration of 10 mM; Table S11: Hydrodynamic diameter and polydispersity index (PDI) of acid-gelled alginate nanoparticles without 5-FU loading (blank) in comparison with Ca^+2^-crosslinked alginate nanoparticles without 5-FU loading (blank) at pH 4.5; Table S12: Hydrodynamic diameter and polydispersity index (PDI) of acid-gelled alginate nanoparticles without 5-FU loading (blank) in comparison with Ca^+2^-crosslinked alginate nanoparticles without 5-FU loading (blank) at pH 10.
