# Supplementary material for: A Green, Solvent- and Cation-Free Approach for Preparing 5-Fluorouracil-Loaded Alginate Nanoparticles Using Microfluidic Technology
Source: Pharmaceutics. 2025 Mar 29;17(4):438. doi: 10.3390/pharmaceutics17040438 (PMC12030154; doi:10.3390/pharmaceutics17040438)
Supplement: Supplementary file 1 [file pharmaceutics-17-00438-s001.zip › pharmaceutics-3482837-supplementary.pdf]

Supplementary Information

Note: All data was analysed using two-way ANOVA (grouped analyses), multiple comparisons, compare column means (main column effect) apart from data in Table S11 (A and B) was analysed using parametric t-test (parametric test), using GraphPad Prism software.

Table S1. Hydrodynamic diameters of acid-gelled alginate nanoparticles unloaded with drug (blank) using hydrochloric acid as a crosslinking agent at different molar concentrations with different pH values; 0.75, 1, and 1.5 respectively.

A: Acid gelled alginate nanoparticles prepared with 0.2% (w/w) low viscosity sodium alginate.

**pH**	0.75	1	1.5
**Size (nm)**	227.9	236.8	825.9
**Size (nm)**	227.9	297.4	481.4
**Size (nm)**	271.6	238.9	531.4

Compare column means (main column effect)								
Number of families	1							
Number of comparisons per family	3							
Alpha	0.05							
Tukey's multiple comparisons test	Mean Diff.	95.00% CI of diff.	Significant?	Summary	Adjusted P Value			
0.75 vs. 1	-15.23	-362.5 to 332.1	No	ns	0.9867			
0.75 vs. 1.5	-370.4	-717.7 to -23.13	Yes	*	0.0408			
1 vs. 1.5	-355.2	-702.5 to -7.894	Yes	*	0.0466			
Test details	Mean 1	Mean 2	Mean Diff.	SE of diff.	N1	N2	q	DF
0.75 vs. 1	242.5	257.7	-15.23	97.45	3	3	0.2211	4.000
0.75 vs. 1.5	242.5	612.9	-370.4	97.45	3	3	5.376	4.000
1 vs. 1.5	257.7	612.9	-355.2	97.45	3	3	5.155	4.000

B: Acid gelled alginate nanoparticles prepared with 0.2% (w/w) high viscosity sodium alginate

**pH**	0.75	1	1.5
**Size (nm)**	274.9	505	409.6
**Size (nm)**	250.3	248.8	921.8
**Size (nm)**	264.4	377.7	351.5

Compare column means (main column effect)								
Number of families	1							
Number of comparisons per family	3							
Alpha	0.05							
Tukey's multiple comparisons test	Mean Diff.	95.00% CI of diff.	Significant?	Summary	Adjusted P Value			
0.75 vs. 1	-114.0	-764.0 to 536.1	No	ns	0.8153			
0.75 vs. 1.5	-297.8	-947.8 to 352.3	No	ns	0.3337			

1 vs. 1.5	-183.8	-833.9 to 466.3	No	ns	0.6111			
Test details	Mean 1	Mean 2	Mean Diff.	SE of diff.	N1	N2	q	DF
0.75 vs. 1	263.2	377.2	-114.0	182.4	3	3	0.8836	4.000
0.75 vs. 1.5	263.2	561.0	-297.8	182.4	3	3	2.309	4.000
1 vs. 1.5	377.2	561.0	-183.8	182.4	3	3	1.425	4.000

C: Acid gelled alginate nanoparticles prepared with 0.4% (w/w) low viscosity sodium alginate at TL.

**pH**	0.75	1	1.5
**Size (nm)**	187	244.1	1379
**Size (nm)**	182.9	352.1	615.2
**Size (nm)**	181.9	224.9	817.8

Compare column means (main column effect)								
Number of families	1							
Number of comparisons per family	3							
Alpha	0.05							
Tukey's multiple comparisons test	Mean Diff.	95.00% CI of diff.	Significant?	Summary	Adjusted P Value			
0.75 vs. 1	-89.77	-795.8 to 616.3	No	ns	0.8958			
0.75 vs. 1.5	-753.4	-1459 to -47.38	Yes	*	0.0408			
1 vs. 1.5	-663.6	-1370 to 42.38	No	ns	0.0604			
Test details	Mean 1	Mean 2	Mean Diff.	SE of diff.	N1	N2	q	DF
0.75 vs. 1	183.9	273.7	-89.77	198.1	3	3	0.6408	4.000
0.75 vs. 1.5	183.9	937.3	-753.4	198.1	3	3	5.379	4.000
1 vs. 1.5	273.7	937.3	-663.6	198.1	3	3	4.738	4.000

D: Acid gelled alginate nanoparticles prepared with 0.4% (w/w) high viscosity sodium alginate.

**pH**	0.75	1	1.5
**Size (nm)**	233	288.3	1689
**Size (nm)**	235.9	291.4	3973
**Size (nm)**	248	256.4	8966

Compare column means (main column effect)								
Number of families	1							
Number of comparisons per family	3							
Alpha	0.05							
Tukey's multiple comparisons test	Mean Diff.	95.00% CI of diff.	Significant?	Summary	Adjusted P Value			
0.75 vs. 1	-39.73	-6301 to 6221	No	ns	0.9997			
0.75 vs. 1.5	-4637	-10898 to 1624	No	ns	0.1183			
1 vs. 1.5	-4597	-10858 to 1664	No	ns	0.1210			
Test details	Mean 1	Mean 2	Mean Diff.	SE of diff.	N1	N2	q	DF
0.75 vs. 1	239.0	278.7	-39.73	1757	3	3	0.03199	4.000
0.75 vs. 1.5	239.0	4876	-4637	1757	3	3	3.733	4.000
1 vs. 1.5	278.7	4876	-4597	1757	3	3	3.701	4.000

Table S2. Polydispersity index (PDI) of acid-gelled alginate nanoparticles unloaded with drug (blank) using hydrochloric acid as a crosslinking agent at different molar concentrations with different pH values; 0.75, 1, and 1.5 respectively.

A: Acid gelled alginate nanoparticles prepared with 0.2% (w/w) low viscosity sodium alginate.

**pH**	0.75	1	1.5
**PDI**	0.307	0.48	0.7382
**PDI**	0.284	0.315	0.4605
**PDI**	0.575	0.513	0.491

Compare column means (main column effect)								
Number of families	1							
Number of comparisons per family	3							
Alpha	0.05							
Tukey's multiple comparisons test	Mean Diff.	95.00% CI of diff.	Significant?	Summary	Adjusted P Value			
0.75 vs. 1	-0.04733	-0.4235 to 0.3288	No	ns	0.8977			
0.75 vs. 1.5	-0.1746	-0.5507 to 0.2016	No	ns	0.3263			
1 vs. 1.5	-0.1272	-0.5034 to 0.2489	No	ns	0.5107			
Test details	Mean 1	Mean 2	Mean Diff.	SE of diff.	N1	N2	q	DF
0.75 vs. 1	0.3887	0.4360	-0.04733	0.1055	3	3	0.6343	4.000
0.75 vs. 1.5	0.3887	0.5632	-0.1746	0.1055	3	3	2.339	4.000
1 vs. 1.5	0.4360	0.5632	-0.1272	0.1055	3	3	1.705	4.000

B: Acid gelled alginate nanoparticles prepared with 0.2% (w/w) high viscosity sodium alginate.

**pH**	0.75	1	1.5
**PDI**	0.414	0.519	0.4544
**PDI**	0.215	0.339	0.7063
**PDI**	0.394	0.398	0.403

Compare column means (main column effect)								
Number of families	1							
Number of comparisons per family	3							
Alpha	0.05							
Tukey's multiple comparisons test	Mean Diff.	95.00% CI of diff.	Significant?	Summary	Adjusted P Value			
0.75 vs. 1	-0.07767	-0.5072 to 0.3519	No	ns	0.8053			
0.75 vs. 1.5	-0.1802	-0.6098 to 0.2493	No	ns	0.3840			
1 vs. 1.5	-0.1026	-0.5321 to 0.3270	No	ns	0.6953			
Test details	Mean 1	Mean 2	Mean Diff.	SE of diff.	N1	N2	q	DF
0.75 vs. 1	0.3410	0.4187	-0.07767	0.1205	3	3	0.9113	4.000
0.75 vs. 1.5	0.3410	0.5212	-0.1802	0.1205	3	3	2.115	4.000
1 vs. 1.5	0.4187	0.5212	-0.1026	0.1205	3	3	1.203	4.000

C: Acid gelled alginate nanoparticles prepared with 0.4% (w/w) low viscosity sodium alginate at TL.

**pH**	0.75	1	1.5
**PDI**	0.305	0.485	1
**PDI**	0.35	0.329	0.6032

**PDI**	0.279	0.412	0.793

Compare column means (main column effect)								
Number of families	1							
Number of comparisons per family	3							
Alpha	0.05							
Tukey's multiple comparisons test	Mean Diff.	95.00% CI of diff.	Significant?	Summary	Adjusted P Value			
0.75 vs. 1	-0.09733	-0.4225 to 0.2279	No	ns	0.5802			
0.75 vs. 1.5	-0.4874	-0.8126 to -0.1622	Yes	*	0.0129			
1 vs. 1.5	-0.3901	-0.7153 to -0.06485	Yes	*	0.0279			
Test details	Mean 1	Mean 2	Mean Diff.	SE of diff.	N1	N2	q	DF
0.75 vs. 1	0.3113	0.4087	-0.09733	0.09125	3	3	1.508	4.000
0.75 vs. 1.5	0.3113	0.7987	-0.4874	0.09125	3	3	7.554	4.000
1 vs. 1.5	0.4087	0.7987	-0.3901	0.09125	3	3	6.045	4.000

D: Acid gelled alginate nanoparticles prepared with 0.4% (w/w) high viscosity sodium alginate.

**pH**	0.75	1	1.5
**PDI**	0.451	0.456	0.4708
**PDI**	0.406	0.574	0.456
**PDI**	0.448	0.535	0.392

Compare column means (main column effect)								
Number of families	1							
Number of comparisons per family	3							
Alpha	0.05							

Tukey's multiple comparisons test	Mean Diff.	95.00% CI of diff.	Significant?	Summary	Adjusted P Value			
0.75 vs. 1	-0.08667	-0.2407 to 0.06740	No	ns	0.2264			
0.75 vs. 1.5	-0.004600	-0.1587 to 0.1495	No	ns	0.9938			
1 vs. 1.5	0.08207	-0.07200 to 0.2361	No	ns	0.2530			
Test details	Mean 1	Mean 2	Mean Diff.	SE of diff.	N1	N2	q	DF
0.75 vs. 1	0.4350	0.5217	-0.08667	0.04323	3	3	2.835	4.000
0.75 vs. 1.5	0.4350	0.4396	-0.004600	0.04323	3	3	0.1505	4.000
1 vs. 1.5	0.5217	0.4396	0.08207	0.04323	3	3	2.685	4.000

Table S3. Zeta potential values of acid-gelled alginate nanoparticles unloaded with drug (blank) using hydrochloric acid as a crosslinking agent at different molar concentrations with different pH values; 0.75, 1, and 1.5 respectively.

A: Acid gelled alginate nanoparticles prepared with 0.2% (w/w) low viscosity sodium alginate.

**pH**	0.75	1	1.5
**Zeta potential (mV)**	-3.649	-0.3625	-4.264
**Zeta potential (mV)**	-0.3064	-4.073	-13.77
**Zeta potential (mV)**	1.287	-7.862	-11.44

Compare column means (main column effect)								
Number of families	1							
Number of comparisons per family	3							
Alpha	0.05							
Tukey's multiple comparisons test	Mean Diff.	95.00% CI of diff.	Significant?	Summary	Adjusted P Value			
0.75 vs. 1	3.210	-8.836 to 15.26	No	ns	0.6420			
0.75 vs. 1.5	8.935	-3.110 to 20.98	No	ns	0.1179			
1 vs. 1.5	5.726	-6.320 to 17.77	No	ns	0.3131			
Test details	Mean 1	Mean 2	Mean Diff.	SE of diff.	N1	N2	q	DF
0.75 vs. 1	-0.8895	-4.099	3.210	3.380	3	3	1.343	4.000
0.75 vs. 1.5	-0.8895	-9.825	8.935	3.380	3	3	3.739	4.000
1 vs. 1.5	-4.099	-9.825	5.726	3.380	3	3	2.396	4.000

B: Acid gelled alginate nanoparticles prepared with 0.2% (w/w) high viscosity sodium alginate.

**pH**	0.75	1	1.5

**Zeta potential (mV)**	-0.7405	0.8664	-5.787
**Zeta potential (mV)**	-0.895	1.364	-4.359
**Zeta potential (mV)**	2.931	1.501	-4.996

Tukey's multiple comparisons test	Mean Diff.	95.00% CI of diff.	Significant?	Summary	Adjusted P Value			
0.75 vs. 1	-0.8120	-4.424 to 2.800	No	ns	0.7223			
0.75 vs. 1.5	5.479	1.867 to 9.091	Yes	*	0.0124			
1 vs. 1.5	6.291	2.679 to 9.903	Yes	**	0.0075			
Test details	Mean 1	Mean 2	Mean Diff.	SE of diff.	N1	N2	q	DF
0.75 vs. 1	0.4318	1.244	-0.8120	1.013	3	3	1.133	4.000
0.75 vs. 1.5	0.4318	-5.047	5.479	1.013	3	3	7.646	4.000
1 vs. 1.5	1.244	-5.047	6.291	1.013	3	3	8.779	4.000

C: Acid gelled alginate nanoparticles prepared with 0.4% (w/w) low viscosity sodium alginate at TL.

**pH**	0.75	1	1.5
**Zeta potential (mV)**	-2.468	-1.078	-5.247
**Zeta potential (mV)**	-3.881	-3.037	-6.893
**Zeta potential (mV)**	-12.04	-3.559	-7.442

Compare column means (main column effect)								
Number of families	1							
Number of comparisons per family	3							
Alpha	0.05							
Tukey's multiple comparisons test	Mean Diff.	95.00% CI of diff.	Significant?	Summary	Adjusted P Value			

0.75 vs. 1	-3.572	-10.79 to 3.646	No	ns	0.2912			
0.75 vs. 1.5	0.3977	-6.820 to 7.615	No	ns	0.9791			
1 vs. 1.5	3.969	-3.248 to 11.19	No	ns	0.2373			
Test details	Mean 1	Mean 2	Mean Diff.	SE of diff.	N1	N2	q	DF
0.75 vs. 1	-6.130	-2.558	-3.572	2.025	3	3	2.494	4.000
0.75 vs. 1.5	-6.130	-6.527	0.3977	2.025	3	3	0.2777	4.000
1 vs. 1.5	-2.558	-6.527	3.969	2.025	3	3	2.772	4.000

D: Acid gelled alginate nanoparticles prepared with 0.4% (w/w) high viscosity sodium alginate.

**pH**	0.75	1	1.5
**Zeta potential (mV)**	-3.965	-12.69	-6.026
**Zeta potential (mV)**	-3.633	-7.307	-5.643
**Zeta potential (mV)**	-3.488	-9.472	-6.415

Compare column means (main column effect)								
Number of families	1							
Number of comparisons per family	3							
Alpha	0.05							
Tukey's multiple comparisons test	Mean Diff.	95.00% CI of diff.	Significant?	Summary	Adjusted P Value			
0.75 vs. 1	6.128	1.792 to 10.46	Yes	*	0.0159			
0.75 vs. 1.5	2.333	-2.003 to 6.668	No	ns	0.2480			
1 vs. 1.5	-3.795	-8.130 to 0.5404	No	ns	0.0746			
Test details	Mean 1	Mean 2	Mean Diff.	SE of diff.	N1	N2	q	DF

0.75 vs. 1	-3.695	-9.823	6.128	1.216	3	3	7.124	4.000
0.75 vs. 1.5	-3.695	-6.028	2.333	1.216	3	3	2.712	4.000
1 vs. 1.5	-9.823	-6.028	-3.795	1.216	3	3	4.412	4.000

Table S4. Hydrodynamic diameters, polydispersity index/PDI and zeta potential values of Ca+2-crosslinked alginate nanoparticles unloaded with 5-FU (Blank).

**Nanoparticle**	A: 0.2 L-ALG (Blank)	B: 0.2 H-ALG (Blank)	C: 0.4 L-ALG (Blank)
**Size (nm)**	711.3	850.3	1474
**Size (nm)**	892.3	817	434.9
**Size (nm)**	874.3	1074	521.1

Compare column means (main column effect)								
Number of families	1							
Number of comparisons per family	6							
Alpha	0.05							
Tukey's multiple comparisons test	Mean Diff.	95.00% CI of diff.	Significant?	Summary	Adjusted P Value			
A: 0.2 L-ALG (Blank) vs. B: 0.2 H-ALG (Blank)	-87.80	-984.2 to 808.6	No	ns	0.9853			
A: 0.2 L-ALG (Blank) vs. C: 0.4 L-ALG (Blank)	15.97	-880.5 to 912.4	No	ns	>0.9999			
A: 0.2 L-ALG (Blank) vs. D: 0.4 H-ALG (Blank)	651.6	-244.8 to 1548	No	ns	0.1541			
B: 0.2 H-ALG (Blank) vs. C: 0.4 L-ALG (Blank)	103.8	-792.7 to 1000	No	ns	0.9763			
B: 0.2 H-ALG (Blank) vs. D: 0.4 H-ALG (Blank)	739.4	-157.0 to 1636	No	ns	0.1023			
C: 0.4 L-ALG (Blank) vs. D: 0.4 H-ALG (Blank)	635.6	-260.8 to 1532	No	ns	0.1660			
Test details	Mean 1	Mean 2	Mean Diff.	SE of diff.	N1	N2	q	DF
A: 0.2 L-ALG (Blank) vs. B: 0.2 H-ALG (Blank)	826.0	913.8	-87.80	259.0	3	3	0.4795	6.000
A: 0.2 L-ALG (Blank) vs. C: 0.4 L-ALG (Blank)	826.0	810.0	15.97	259.0	3	3	0.08720	6.000
A: 0.2 L-ALG (Blank) vs. D: 0.4 H-ALG (Blank)	826.0	174.4	651.6	259.0	3	3	3.559	6.000
B: 0.2 H-ALG (Blank) vs. C: 0.4 L-ALG (Blank)	913.8	810.0	103.8	259.0	3	3	0.5667	6.000
B: 0.2 H-ALG (Blank) vs. D: 0.4 H-ALG (Blank)	913.8	174.4	739.4	259.0	3	3	4.038	6.000
C: 0.4 L-ALG (Blank) vs. D: 0.4 H-ALG (Blank)	810.0	174.4	635.6	259.0	3	3	3.471	6.000

**Nanoparticle**	A: 0.2 L-ALG (Blank)	B: 0.2 H-ALG (Blank)	C: 0.4 L-ALG (Blank)

**PDI**	0.49	0.681	0.964
**PDI**	0.408	0.664	0.443
**PDI**	0.652	0.766	0.527

Compare column means (main column effect)								
Number of families	1							
Number of comparisons per family	6							
Alpha	0.05							
Tukey's multiple comparisons test	Mean Diff.	95.00% CI of diff.	Significant?	Summary	Adjusted P Value			
A: 0.2 L-ALG (Blank) vs. B: 0.2 H-ALG (Blank)	-0.1870	-0.7512 to 0.3772	No	ns	0.6771			
A: 0.2 L-ALG (Blank) vs. C: 0.4 L-ALG (Blank)	-0.1280	-0.6922 to 0.4362	No	ns	0.8587			
A: 0.2 L-ALG (Blank) vs. D: 0.4 H-ALG (Blank)	0.07533	-0.4889 to 0.6395	No	ns	0.9647			
B: 0.2 H-ALG (Blank) vs. C: 0.4 L-ALG (Blank)	0.05900	-0.5052 to 0.6232	No	ns	0.9823			
B: 0.2 H-ALG (Blank) vs. D: 0.4 H-ALG (Blank)	0.2623	-0.3019 to 0.8265	No	ns	0.4396			
C: 0.4 L-ALG (Blank) vs. D: 0.4 H-ALG (Blank)	0.2033	-0.3609 to 0.7675	No	ns	0.6230			
Test details	Mean 1	Mean 2	Mean Diff.	SE of diff.	N1	N2	q	DF
A: 0.2 L-ALG (Blank) vs. B: 0.2 H-ALG (Blank)	0.5167	0.7037	-0.1870	0.1630	3	3	1.623	6.000
A: 0.2 L-ALG (Blank) vs. C: 0.4 L-ALG (Blank)	0.5167	0.6447	-0.1280	0.1630	3	3	1.111	6.000
A: 0.2 L-ALG (Blank) vs. D: 0.4 H-ALG (Blank)	0.5167	0.4413	0.07533	0.1630	3	3	0.6537	6.000
B: 0.2 H-ALG (Blank) vs. C: 0.4 L-ALG (Blank)	0.7037	0.6447	0.05900	0.1630	3	3	0.5120	6.000
B: 0.2 H-ALG (Blank) vs. D: 0.4 H-ALG (Blank)	0.7037	0.4413	0.2623	0.1630	3	3	2.276	6.000
C: 0.4 L-ALG (Blank) vs. D: 0.4 H-ALG (Blank)	0.6447	0.4413	0.2033	0.1630	3	3	1.764	6.000

**Nanoparticle**	A: 0.2 L-ALG (Blank)	B: 0.2 H-ALG (Blank)	C: 0.4 L-ALG (Blank)
**Zeta potential (mV)**	-23.95	-21.05	-23.65
**Zeta potential (mV)**	-25.2	-22.41	-21.64

**Zeta potential (mV)**	-25.36	-21.48	-25.08

Compare column means (main column effect)								
Number of families	1							
Number of comparisons per family	6							
Alpha	0.05							
Tukey's multiple comparisons test	Mean Diff.	95.00% CI of diff.	Significant?	Summary	Adjusted P Value			
A: 0.2 L-ALG (Blank) vs. B: 0.2 H-ALG (Blank)	-3.190	-6.551 to 0.1707	No	ns	0.0613			
A: 0.2 L-ALG (Blank) vs. C: 0.4 L-ALG (Blank)	-1.380	-4.741 to 1.981	No	ns	0.5314			
A: 0.2 L-ALG (Blank) vs. D: 0.4 H-ALG (Blank)	3.037	-0.3240 to 6.397	No	ns	0.0739			
B: 0.2 H-ALG (Blank) vs. C: 0.4 L-ALG (Blank)	1.810	-1.551 to 5.171	No	ns	0.3328			
B: 0.2 H-ALG (Blank) vs. D: 0.4 H-ALG (Blank)	6.227	2.866 to 9.587	Yes	**	0.0028			
C: 0.4 L-ALG (Blank) vs. D: 0.4 H-ALG (Blank)	4.417	1.056 to 7.777	Yes	*	0.0153			
Test details	Mean 1	Mean 2	Mean Diff.	SE of diff.	N1	N2	q	DF
A: 0.2 L-ALG (Blank) vs. B: 0.2 H-ALG (Blank)	-24.84	-21.65	-3.190	0.9708	3	3	4.647	6.000
A: 0.2 L-ALG (Blank) vs. C: 0.4 L-ALG (Blank)	-24.84	-23.46	-1.380	0.9708	3	3	2.010	6.000
A: 0.2 L-ALG (Blank) vs. D: 0.4 H-ALG (Blank)	-24.84	-27.87	3.037	0.9708	3	3	4.424	6.000
B: 0.2 H-ALG (Blank) vs. C: 0.4 L-ALG (Blank)	-21.65	-23.46	1.810	0.9708	3	3	2.637	6.000
B: 0.2 H-ALG (Blank) vs. D: 0.4 H-ALG (Blank)	-21.65	-27.87	6.227	0.9708	3	3	9.070	6.000
C: 0.4 L-ALG (Blank) vs. D: 0.4 H-ALG (Blank)	-23.46	-27.87	4.417	0.9708	3	3	6.434	6.000

Table S5. Hydrodynamic diameters of acid-gelled alginate nanoparticles loaded with 5-FU at different theoretical loadings (TLs) 34% and 81% using hydrochloric acid as a crosslinking agent at different molar concentrations with different pH values; 0.75, 1, and 1.5 respectively.

A: Acid gelled alginate nanoparticles prepared with 0.2% (w/w) low viscosity sodium alginate at TL of 81%.

**pH**	0.75	1	1.5
**Size (nm)**	883	1118	1692
**Size (nm)**	548.5	1383	1571
**Size (nm)**	577.2	728.2	1459

Compare column means (main column effect)								
Number of families	1							
Number of comparisons per family	3							
Alpha	0.05							
Tukey's multiple comparisons test	Mean Diff.	95.00% CI of diff.	Significant?	Summary	Adjusted P Value			
0.75 vs. 1	-406.8	-975.0 to 161.4	No	ns	0.1292			
0.75 vs. 1.5	-904.4	-1473 to -336.2	Yes	*	0.0104			
1 vs. 1.5	-497.6	-1066 to 70.60	No	ns	0.0745			
Test details	Mean 1	Mean 2	Mean Diff.	SE of diff.	N1	N2	q	DF

0.75 vs. 1	669.6	1076	-406.8	159.4	3	3	3.609	4.000
0.75 vs. 1.5	669.6	1574	-904.4	159.4	3	3	8.023	4.000
1 vs. 1.5	1076	1574	-497.6	159.4	3	3	4.414	4.000

B: Acid gelled nanoparticles prepared with 0.2% (w/w) high viscosity sodium alginate at TL of 81%.

**pH**	1	0.75	1.5
**Size (nm)**	574.9	673.9	1161
**Size (nm)**	957.9	783.3	1208
**Size (nm)**	592	449.7	1360

Compare column means (main column effect)								
Number of families	1							
Number of comparisons per family	3							
Alpha	0.05							
Tukey's multiple comparisons test	Mean Diff.	95.00% CI of diff.	Significant?	Summary	Adjusted P Value			
1 vs. 0.75	72.63	-404.3 to 549.6	No	ns	0.8555			
1 vs. 1.5	-534.7	-1012 to -57.77	Yes	*	0.0348			
0.75 vs. 1.5	-607.4	-1084 to -130.4	Yes	*	0.0228			
Test details	Mean 1	Mean 2	Mean Diff.	SE of diff.	N1	N2	q	DF
1 vs. 0.75	708.3	635.6	72.63	133.8	3	3	0.7675	4.000
1 vs. 1.5	708.3	1243	-534.7	133.8	3	3	5.651	4.000
0.75 vs. 1.5	635.6	1243	-607.4	133.8	3	3	6.418	4.000

C: Acid gelled alginate nanoparticles prepared with 0.4% (w/w) low viscosity sodium alginate at TL of 34%.

**pH**	0.75	1	1.5

**Size (nm)**	562.7	565.3	1845
**Size (nm)**	658.1	900.2	1977
**Size (nm)**	502.9	542.9	1002

Compare column means (main column effect)								
Number of families	1							
Number of comparisons per family	3							
Alpha	0.05							
Tukey's multiple comparisons test	Mean Diff.	95.00% CI of diff.	Significant?	Summary	Adjusted P Value			
0.75 vs. 1	-94.90	-859.1 to 669.3	No	ns	0.9002			
0.75 vs. 1.5	-1033	-1798 to -269.3	Yes	*	0.0185			
1 vs. 1.5	-938.5	-1703 to -174.4	Yes	*	0.0257			
Test details	Mean 1	Mean 2	Mean Diff.	SE of diff.	N1	N2	q	DF
0.75 vs. 1	574.6	669.5	-94.90	214.4	3	3	0.6259	4.000
0.75 vs. 1.5	574.6	1608	-1033	214.4	3	3	6.816	4.000
1 vs. 1.5	669.5	1608	-938.5	214.4	3	3	6.190	4.000

D: Acid-gelled alginate nanoparticles prepared with 0.4% (w/w) high viscosity sodium alginate at TL of 34%.

**pH**	0.75	1	1.5
**Size (nm)**	575	579.1	2488
**Size (nm)**	562.6	603.9	2570
**Size (nm)**	556.2	471.3	1426

Compare column means (main column effect)								
Number of families	1							
Number of comparisons per family	3							

Alpha	0.05							
Tukey's multiple comparisons test	Mean Diff.	95.00% CI of diff.	Significant?	Summary	Adjusted P Value			
0.75 vs. 1	13.17	-998.8 to 1025	No	ns	0.9988			
0.75 vs. 1.5	-1597	-2609 to -584.8	Yes	*	0.0108			
1 vs. 1.5	-1610	-2622 to -598.0	Yes	*	0.0105			
Test details	Mean 1	Mean 2	Mean Diff.	SE of diff.	N1	N2	q	DF
0.75 vs. 1	564.6	551.4	13.17	283.9	3	3	0.06558	4.000
0.75 vs. 1.5	564.6	2161	-1597	283.9	3	3	7.953	4.000
1 vs. 1.5	551.4	2161	-1610	283.9	3	3	8.019	4.000

Table S6. Polydispersity index (PDI) of acid-gelled alginate nanoparticles loaded with 5-FU at different theoretical loadings 34% and 81% using hydrochloric acid as a crosslinking agent at different molar concentrations with different pH values; 0.75, 1, and 1.5 respectively.

A: Acid gelled alginate nanoparticles prepared with 0.2% (w/w) low viscosity sodium alginate at TL of 81%.

**pH**	0.75	1	1.5
**PDI**	0.6295	0.8254	0.9596
**PDI**	0.573	1	0.851
**PDI**	0.477	0.5368	0.7299

Compare column means (main column effect)								
Number of families	1							
Number of comparisons per family	3							
Alpha	0.05							
Tukey's multiple comparisons test	Mean Diff.	95.00% CI of diff.	Significant?	Summary	Adjusted P Value			
0.75 vs. 1	-0.2276	-0.5407 to 0.08554	No	ns	0.1243			
0.75 vs. 1.5	-0.2870	-0.6001 to 0.02610	No	ns	0.0651			
1 vs. 1.5	-0.05943	-0.3725 to 0.2537	No	ns	0.7887			
Test details	Mean 1	Mean 2	Mean Diff.	SE of diff.	N1	N2	q	DF
0.75 vs. 1	0.5598	0.7874	-0.2276	0.08785	3	3	3.663	4.000
0.75 vs. 1.5	0.5598	0.8468	-0.2870	0.08785	3	3	4.620	4.000
1 vs. 1.5	0.7874	0.8468	-0.05943	0.08785	3	3	0.9567	4.000

B: Acid gelled nanoparticles prepared with 0.2% (w/w) high viscosity sodium alginate at TL of 81%.

**pH**	0.75	1	1.5
**PDI**	0.4905	0.4954	1

**PDI**	0.5412	0.5651	0.9497
**PDI**	0.4905	0.5638	0.8228

Compare column means (main column effect)								
Number of families	1							
Number of comparisons per family	3							
Alpha	0.05							
Tukey's multiple comparisons test	Mean Diff.	95.00% CI of diff.	Significant?	Summary	Adjusted P Value			
0.75 vs. 1	-0.03403	-0.2190 to 0.1509	No	ns	0.7994			
0.75 vs. 1.5	-0.4168	-0.6017 to -0.2318	Yes	**	0.0029			
1 vs. 1.5	-0.3827	-0.5677 to -0.1978	Yes	**	0.0040			
Test details	Mean 1	Mean 2	Mean Diff.	SE of diff.	N1	N2	q	DF
0.75 vs. 1	0.5074	0.5414	-0.03403	0.05189	3	3	0.9276	4.000
0.75 vs. 1.5	0.5074	0.9242	-0.4168	0.05189	3	3	11.36	4.000
1 vs. 1.5	0.5414	0.9242	-0.3827	0.05189	3	3	10.43	4.000

C: Acid gelled alginate nanoparticles prepared with 0.4% (w/w) low viscosity sodium alginate at TL of 34%.

**pH**	0.75	1	1.5
**PDI**	0.5378	0.5315	1
**PDI**	0.5594	0.6263	1
**PDI**	0.5477	0.6148	0.5896

Compare column means (main column effect)								

Number of families	1							
Number of comparisons per family	3							
Alpha	0.05							
Tukey's multiple comparisons test	Mean Diff.	95.00% CI of diff.	Significant?	Summary	Adjusted P Value			
0.75 vs. 1	-0.04257	-0.4650 to 0.3799	No	ns	0.9325			
0.75 vs. 1.5	-0.3149	-0.7373 to 0.1075	No	ns	0.1164			
1 vs. 1.5	-0.2723	-0.6948 to 0.1501	No	ns	0.1672			
Test details	Mean 1	Mean 2	Mean Diff.	SE of diff.	N1	N2	q	DF
0.75 vs. 1	0.5483	0.5909	-0.04257	0.1185	3	3	0.5079	4.000
0.75 vs. 1.5	0.5483	0.8632	-0.3149	0.1185	3	3	3.757	4.000
1 vs. 1.5	0.5909	0.8632	-0.2723	0.1185	3	3	3.249	4.000

D: Acid gelled-alginate nanoparticles prepared with 0.4% (w/w) high viscosity sodium alginate at TL of 34%.

**pH**	0.75	1	1.5
**PDI**	1	0.4728	1
**PDI**	0.4672	0.4978	1
**PDI**	0.486	0.5815	0.8267

Compare column means (main column effect)								
Number of families	1							
Number of comparisons per family	3							
Alpha	0.05							
Tukey's multiple comparisons test	Mean Diff.	95.00% CI of diff.	Significant?	Summary	Adjusted P Value			
0.75 vs. 1	0.1337	-0.4163 to 0.6837	No	ns	0.6870			
0.75 vs. 1.5	-0.2912	-0.8412 to 0.2589	No	ns	0.2561			
1 vs. 1.5	-0.4249	-0.9749 to 0.1252	No	ns	0.1058			
Test details	Mean 1	Mean 2	Mean Diff.	SE of diff.	N1	N2	q	DF
0.75 vs. 1	0.6511	0.5174	0.1337	0.1543	3	3	1.225	4.000
0.75 vs. 1.5	0.6511	0.9422	-0.2912	0.1543	3	3	2.668	4.000

1 vs. 1.5	0.5174	0.9422	-0.4249	0.1543	3	3	3.893	4.000

Table S7. Zeta potential of acid-gelled alginate nanoparticles loaded with 5-FU at different theoretical loadings (TLs) 34% and 81% using hydrochloric acid as a crosslinking agent at different molar concentrations with different pH values; 0.75, 1, and 1.5 respectively.

A: Acid gelled alginate nanoparticles prepared with 0.2% (w/w) low viscosity sodium alginate at TL of 81%.

**pH**	0.75	1	1.5
**Zeta potential (Mv)**	-7.018	-2.938	-4.919
**Zeta potential (Mv)**	0.5702	-6.021	-5.131
**Zeta potential (Mv)**	-1.466	-18.11	-6.217

Compare column means (main column effect)								
Number of families	1							
Number of comparisons per family	3							
Alpha	0.05							
Tukey's multiple comparisons test	Mean Diff.	95.00% CI of diff.	Significant?	Summary	Adjusted P Value			
0.75 vs. 1	6.385	-9.516 to 22.29	No	ns	0.4097			
0.75 vs. 1.5	2.784	-13.12 to 18.69	No	ns	0.8157			
1 vs. 1.5	-3.601	-19.50 to 12.30	No	ns	0.7191			
Test details	Mean 1	Mean 2	Mean Diff.	SE of diff.	N1	N2	q	DF
0.75 vs. 1	-2.638	-9.023	6.385	4.462	3	3	2.024	4.000
0.75 vs. 1.5	-2.638	-5.422	2.784	4.462	3	3	0.8826	4.000
1 vs. 1.5	-9.023	-5.422	-3.601	4.462	3	3	1.141	4.000

B: Acid gelled nanoparticles prepared with 0.2% (w/w) high viscosity sodium alginate at TL of 81%.

**pH**	0.75	1	1.5
**Zeta potential (Mv)**	-6.376	-1.89	-5.769
**Zeta potential (Mv)**	-14.31	0.6114	-5.716

**Zeta potential (Mv)**	-6.988	-1.464	-5.912

Compare column means (main column effect)								
Number of families	1							
Number of comparisons per family	3							
Alpha	0.05							
Tukey's multiple comparisons test	Mean Diff.	95.00% CI of diff.	Significant?	Summary	Adjusted P Value			
0.75 vs. 1	-8.310	-17.10 to 0.4822	No	ns	0.0594			
0.75 vs. 1.5	-3.426	-12.22 to 5.367	No	ns	0.4275			
1 vs. 1.5	4.885	-3.908 to 13.68	No	ns	0.2324			
Test details	Mean 1	Mean 2	Mean Diff.	SE of diff.	N1	N2	q	DF
0.75 vs. 1	-9.225	-0.9142	-8.310	2.467	3	3	4.764	4.000
0.75 vs. 1.5	-9.225	-5.799	-3.426	2.467	3	3	1.964	4.000
1 vs. 1.5	-0.9142	-5.799	4.885	2.467	3	3	2.800	4.000

C: Acid gelled alginate nanoparticles prepared with 0.4% (w/w) low viscosity sodium alginate at TL of 34%.

**pH**	0.75	1	1.5
**Zeta potential (Mv)**	-6.669	-4.67	-5.163
**Zeta potential (Mv)**	-4.87	-3.88	-5.15
**Zeta potential (Mv)**	-6.728	-7.035	-5.716

Compare column means (main column effect)								
Number of families	1							
Number of comparisons per family	3							
Alpha	0.05							
Tukey's multiple comparisons test	Mean Diff.	95.00% CI of diff.	Significant?	Summary	Adjusted P Value			

0.75 vs. 1	-0.8940	-3.256 to 1.468	No	ns	0.4445			
0.75 vs. 1.5	-0.7460	-3.108 to 1.616	No	ns	0.5500			
1 vs. 1.5	0.1480	-2.214 to 2.510	No	ns	0.9731			
Test details	Mean 1	Mean 2	Mean Diff.	SE of diff.	N1	N2	q	DF
0.75 vs. 1	-6.089	-5.195	-0.8940	0.6626	3	3	1.908	4.000
0.75 vs. 1.5	-6.089	-5.343	-0.7460	0.6626	3	3	1.592	4.000
1 vs. 1.5	-5.195	-5.343	0.1480	0.6626	3	3	0.3159	4.000

D: Acid gelled-alginate nanoparticles prepared with 0.4% (w/w) high viscosity sodium alginate at TL of 34%.

**pH**	0.75	1	1.5
**Zeta potential (Mv)**	-9.302	-8.402	-5.815
**Zeta potential (Mv)**	2.911	-13	-5.508
**Zeta potential (Mv)**	-11.14	0.7516	-6.796

Compare column means (main column effect)								
Number of families	1							
Number of comparisons per family	3							
Alpha	0.05							
Tukey's multiple comparisons test	Mean Diff.	95.00% CI of diff.	Significant?	Summary	Adjusted P Value			
0.75 vs. 1	1.040	-19.73 to 21.81	No	ns	0.9827			
0.75 vs. 1.5	0.1960	-20.58 to 20.97	No	ns	0.9994			
1 vs. 1.5	-0.8438	-21.62 to 19.93	No	ns	0.9885			
Test details	Mean 1	Mean 2	Mean Diff.	SE of diff.	N1	N2	q	DF
0.75 vs. 1	-5.844	-6.883	1.040	5.829	3	3	0.2523	4.000
0.75 vs. 1.5	-5.844	-6.040	0.1960	5.829	3	3	0.04755	4.000
1 vs. 1.5	-6.883	-6.040	-0.8438	5.829	3	3	0.2047	4.000

Table S8. Hydrodynamic diameters, polydispersity index/PDI and zeta potential values of Ca+2-crosslinked alginate nanoparticles loaded with 5-FU at different theoretical loadings 34% and 81% using calcium chloride as a crosslinking agent at different molar concentration of 10 mM.

**Nanoparticle**	A: 0.2 L-ALG (TL%=81)	B: 0.2 H-ALG (TL%=81)	C: 0.4 L-ALG (TL%=34)	D: 0.4 H-ALG (TL%=34)
**Size (nm)**	888.4	2502	995.9	1275
**Size (nm)**	681.4	1046	687.7	832.9
**Size (nm)**	582.6	665.2	434.6	863.6

Compare column means (main column effect)									
Number of families	1								
Number of comparisons per family	6								
Alpha	0.05								
Tukey's multiple comparisons test	Mean Diff.	95.00% CI of diff.	Significant?	Summary	Adjusted P Value				
A: 0.2 L-ALG (TL%=81) vs. B: 0.2 H-ALG (TL%=81)	-686.9	-1765 to 391.2	No	ns	0.2238				
A: 0.2 L-ALG (TL%=81) vs. C: 0.4 L-ALG (TL%=34)	11.40	-1067 to 1090	No	ns	>0.9999				
A: 0.2 L-ALG (TL%=81) vs. D: 0.4 H-ALG (TL%=34)	-273.0	-1351 to 805.1	No	ns	0.8170				
B: 0.2 H-ALG (TL%=81) vs. C: 0.4 L-ALG (TL%=34)	698.3	-379.8 to 1777	No	ns	0.2142				
B: 0.2 H-ALG (TL%=81) vs. D: 0.4 H-ALG (TL%=34)	413.9	-664.3 to 1492	No	ns	0.5796				
C: 0.4 L-ALG (TL%=34) vs. D: 0.4 H-ALG (TL%=34)	-284.4	-1363 to 793.7	No	ns	0.7993				
Test details	Mean 1	Mean 2	Mean Diff.	SE of diff.	N1	N2	q	DF	
A: 0.2 L-ALG (TL%=81) vs. B: 0.2 H-ALG (TL%=81)	717.5	1404	-686.9	311.5	3	3	3.119	6.000	
A: 0.2 L-ALG (TL%=81) vs. C: 0.4 L-ALG (TL%=34)	717.5	706.1	11.40	311.5	3	3	0.05176	6.000	
A: 0.2 L-ALG (TL%=81) vs. D: 0.4 H-ALG (TL%=34)	717.5	990.5	-273.0	311.5	3	3	1.240	6.000	
B: 0.2 H-ALG (TL%=81) vs. C: 0.4 L-ALG (TL%=34)	1404	706.1	698.3	311.5	3	3	3.171	6.000	
B: 0.2 H-ALG (TL%=81) vs. D: 0.4 H-ALG (TL%=34)	1404	990.5	413.9	311.5	3	3	1.879	6.000	
C: 0.4 L-ALG (TL%=34) vs. D: 0.4 H-ALG (TL%=34)	706.1	990.5	-284.4	311.5	3	3	1.292	6.000	

**Nanoparticle**	A: 0.2 L-ALG (TL%=81)	B: 0.2 H-ALG (TL%=81)	C: 0.4 L-ALG (TL%=34)	D: 0.4 H-ALG (TL%=34)
**PDI**	0.6498	1	0.7326	0.9013
**PDI**	0.4817	0.6747	0.4885	0.6976
**PDI**	0.4962	0.4819	0.4734	0.6018

Compare column means (main column effect)								
Number of families	1							
Number of comparisons per family	6							
Alpha	0.05							
Tukey's multiple comparisons test	Mean Diff.	95.00% CI of diff.	Significant?	Summary	Adjusted P Value			
A: 0.2 L-ALG (TL%=81) vs. B: 0.2 H-ALG (TL%=81)	-0.1763	-0.3973 to 0.04466	No	ns	0.1145			
A: 0.2 L-ALG (TL%=81) vs. C: 0.4 L-ALG (TL%=34)	-0.02227	-0.2432 to 0.1987	No	ns	0.9840			
A: 0.2 L-ALG (TL%=81) vs. D: 0.4 H-ALG (TL%=34)	-0.1910	-0.4120 to 0.02996	No	ns	0.0868			
B: 0.2 H-ALG (TL%=81) vs. C: 0.4 L-ALG (TL%=34)	0.1540	-0.06693 to 0.3750	No	ns	0.1745			
B: 0.2 H-ALG (TL%=81) vs. D: 0.4 H-ALG (TL%=34)	-0.01470	-0.2357 to 0.2063	No	ns	0.9952			
C: 0.4 L-ALG (TL%=34) vs. D: 0.4 H-ALG (TL%=34)	-0.1687	-0.3897 to 0.05223	No	ns	0.1321			
Test details	Mean 1	Mean 2	Mean Diff.	SE of diff.	N1	N2	q	DF
A: 0.2 L-ALG (TL%=81) vs. B: 0.2 H-ALG (TL%=81)	0.5426	0.7189	-0.1763	0.06383	3	3	3.906	6.000
A: 0.2 L-ALG (TL%=81) vs. C: 0.4 L-ALG (TL%=34)	0.5426	0.5648	-0.02227	0.06383	3	3	0.4933	6.000
A: 0.2 L-ALG (TL%=81) vs. D: 0.4 H-ALG (TL%=34)	0.5426	0.7336	-0.1910	0.06383	3	3	4.232	6.000
B: 0.2 H-ALG (TL%=81) vs. C: 0.4 L-ALG (TL%=34)	0.7189	0.5648	0.1540	0.06383	3	3	3.413	6.000
B: 0.2 H-ALG (TL%=81) vs. D: 0.4 H-ALG (TL%=34)	0.7189	0.7336	-0.01470	0.06383	3	3	0.3257	6.000
C: 0.4 L-ALG (TL%=34) vs. D: 0.4 H-ALG (TL%=34)	0.5648	0.7336	-0.1687	0.06383	3	3	3.738	6.000

**Nanoparticle**	A: 0.2 L-ALG (TL%=81)	B: 0.2 H-ALG (TL%=81)	C: 0.4 L-ALG (TL%=34)	D: 0.4 H-ALG (TL%=34)
**Zeta potential (mV)**	-23.68	-18.47	-22.96	-21.78

**Zeta potential (mV)**	-21.68	-19.61	-21.78	-24.46
**Zeta potential (mV)**	-22.41	-17.85	-27.94	-27.5

Compare column means (main column effect)								
Number of families	1							
Number of comparisons per family	6							
Alpha	0.05							
Tukey's multiple comparisons test	Mean Diff.	95.00% CI of diff.	Significant?	Summary	Adjusted P Value			
A: 0.2 L-ALG (TL%=81) vs. B: 0.2 H-ALG (TL%=81)	-3.947	-10.20 to 2.303	No	ns	0.2291			
A: 0.2 L-ALG (TL%=81) vs. C: 0.4 L-ALG (TL%=34)	1.637	-4.613 to 7.887	No	ns	0.8026			
A: 0.2 L-ALG (TL%=81) vs. D: 0.4 H-ALG (TL%=34)	1.990	-4.260 to 8.240	No	ns	0.7014			
B: 0.2 H-ALG (TL%=81) vs. C: 0.4 L-ALG (TL%=34)	5.583	-0.6668 to 11.83	No	ns	0.0771			
B: 0.2 H-ALG (TL%=81) vs. D: 0.4 H-ALG (TL%=34)	5.937	-0.3134 to 12.19	No	ns	0.0612			
C: 0.4 L-ALG (TL%=34) vs. D: 0.4 H-ALG (TL%=34)	0.3533	-5.897 to 6.603	No	ns	0.9971			
Test details	Mean 1	Mean 2	Mean Diff.	SE of diff.	N1	N2	q	DF
A: 0.2 L-ALG (TL%=81) vs. B: 0.2 H-ALG (TL%=81)	-22.59	-18.64	-3.947	1.805	3	3	3.091	6.000
A: 0.2 L-ALG (TL%=81) vs. C: 0.4 L-ALG (TL%=34)	-22.59	-24.23	1.637	1.805	3	3	1.282	6.000
A: 0.2 L-ALG (TL%=81) vs. D: 0.4 H-ALG (TL%=34)	-22.59	-24.58	1.990	1.805	3	3	1.559	6.000
B: 0.2 H-ALG (TL%=81) vs. C: 0.4 L-ALG (TL%=34)	-18.64	-24.23	5.583	1.805	3	3	4.373	6.000
B: 0.2 H-ALG (TL%=81) vs. D: 0.4 H-ALG (TL%=34)	-18.64	-24.58	5.937	1.805	3	3	4.650	6.000
C: 0.4 L-ALG (TL%=34) vs. D: 0.4 H-ALG (TL%=34)	-24.23	-24.58	0.3533	1.805	3	3	0.2768	6.000

Table S9. Encapsulation efficiency (EE%) of 5-FU loaded into acid-gelled alginate nanoparticles at different theoretical loadings (TLs) with 34% and 81%.

A: Acid-gelled alginate nanoparticles prepared with 0.2% (w/w) low viscosity sodium alginate at TL of 81%. B: Acid-gelled

**pH**	0.75	1	1.5
**Encapsulation efficiency (EE%)**	12.2	15.45	21.27
**Encapsulation efficiency (EE%)**	12.63	15.72	14.22
**Encapsulation efficiency (EE%)**	12.66	15.87	18.1

Compare column means (main column effect)								
Number of families	1							
Number of comparisons per family	3							
Alpha	0.05							
Tukey's multiple comparisons test	Mean Diff.	95.00% CI of diff.	Significant?	Summary	Adjusted P Value			
0.75 vs. 1	-3.183	-9.401 to 3.035	No	ns	0.2733			
0.75 vs. 1.5	-5.367	-11.58 to 0.8514	No	ns	0.0777			
1 vs. 1.5	-2.183	-8.401 to 4.035	No	ns	0.4889			
Test details	Mean 1	Mean 2	Mean Diff.	SE of diff.	N1	N2	q	DF
0.75 vs. 1	12.50	15.68	-3.183	1.745	3	3	2.580	4.000
0.75 vs. 1.5	12.50	17.86	-5.367	1.745	3	3	4.350	4.000
1 vs. 1.5	15.68	17.86	-2.183	1.745	3	3	1.770	4.000

B: Acid-gelled alginate nanoparticles prepared with 0.2% (w/w) high viscosity sodium alginate at TL of 81%.

**pH**	0.75	1	1.5

**Encapsulation efficiency (EE%)**	16.44	17.01	18.74
**Encapsulation efficiency (EE%)**	16.36	18.31	20.54
**Encapsulation efficiency (EE%)**	16.04	17.88	20.61

Compare column means (main column effect)								
Number of families	1							
Number of comparisons per family	3							
Alpha	0.05							
Tukey's multiple comparisons test	Mean Diff.	95.00% CI of diff.	Significant?	Summary	Adjusted P Value			
0.75 vs. 1	-1.453	-3.260 to 0.3528	No	ns	0.0947			
0.75 vs. 1.5	-3.683	-5.490 to -1.877	Yes	**	0.0042			
1 vs. 1.5	-2.230	-4.036 to -0.4238	Yes	*	0.0253			
Test details	Mean 1	Mean 2	Mean Diff.	SE of diff.	N1	N2	q	DF
0.75 vs. 1	16.28	17.73	-1.453	0.5068	3	3	4.056	4.000
0.75 vs. 1.5	16.28	19.96	-3.683	0.5068	3	3	10.28	4.000
1 vs. 1.5	17.73	19.96	-2.230	0.5068	3	3	6.223	4.000

C: Acid-gelled alginate nanoparticles prepared with 0.4% (w/w) low viscosity sodium alginate at TL of 34%.

**pH**	0.75	1	1.5
**Encapsulation efficiency (EE%)**	10.59	10.21	23.93
**Encapsulation efficiency (EE%)**	15.09	10.97	21.5
**Encapsulation efficiency (EE%)**	15.09	11.73	19.01

Compare column means (main column effect)								
Number of families	1							
Number of comparisons per family	3							

Alpha	0.05							
Tukey's multiple comparisons test	Mean Diff.	95.00% CI of diff.	Significant?	Summary	Adjusted P Value			
0.75 vs. 1	2.620	-4.713 to 9.953	No	ns	0.4786			
0.75 vs. 1.5	-7.890	-15.22 to -0.5568	Yes	*	0.0397			
1 vs. 1.5	-10.51	-17.84 to -3.177	Yes	*	0.0151			
Test details	Mean 1	Mean 2	Mean Diff.	SE of diff.	N1	N2	q	DF
0.75 vs. 1	13.59	10.97	2.620	2.058	3	3	1.801	4.000
0.75 vs. 1.5	13.59	21.48	-7.890	2.058	3	3	5.423	4.000
1 vs. 1.5	10.97	21.48	-10.51	2.058	3	3	7.224	4.000

D: Acid-gelled alginate nanoparticles prepared with 0.4% (w/w) high viscosity sodium alginate at TL of 34%.

pH	0.75	1	1.5
Encapsulation efficiency (EE%)	29.17	21.49	24.7
Encapsulation efficiency (EE%)	30.69	21.94	24.83
Encapsulation efficiency (EE%)	29.48	19.47	37

Compare column means (main column effect)								
Number of families	1							
Number of comparisons per family	3							
Alpha	0.05							
Tukey's multiple comparisons test	Mean Diff.	95.00% CI of diff.	Significant?	Summary	Adjusted P Value			
0.75 vs. 1	8.813	-4.490 to 22.12	No	ns	0.1566			
0.75 vs. 1.5	0.9367	-12.37 to 14.24	No	ns	0.9662			
1 vs. 1.5	-7.877	-21.18 to 5.427	No	ns	0.2029			
Test details	Mean 1	Mean 2	Mean Diff.	SE of diff.	N1	N2	q	DF
0.75 vs. 1	29.78	20.97	8.813	3.733	3	3	3.339	4.000
0.75 vs. 1.5	29.78	28.84	0.9367	3.733	3	3	0.3549	4.000
1 vs. 1.5	20.97	28.84	-7.877	3.733	3	3	2.984	4.000

Table S10. Encapsulation efficiency (EE%) of 5-FU loaded Ca+2-crosslinked alginate nanoparticles at different theoretical loadings (TLs) with 34% and 81% using calcium chloride as a crosslinking agent at molar concentration of 10 mM.

**Nanoparticle**	A: 0.2 L-ALG (TL%=81)	B: 0.2 H-ALG (TL%=81)	C: 0.4 L-ALG (TL%=34)	D: 0.4 H-ALG (TL%=34)
Encapsulation efficiency (EE%)	17.96	8.526	27.79	39.03
Encapsulation efficiency (EE%)	11.52	15.17	29.47	38.45
Encapsulation efficiency (EE%)	16.29	9.973	29.86	39.74

Compare column means (main column effect)								
Number of families	1							
Number of comparisons per family	6							
Alpha	0.05							
Tukey's multiple comparisons test	Mean Diff.	95.00% CI of diff.	Significant?	Summary	Adjusted P Value			
A: 0.2 L-ALG (TL%=81) vs. B: 0.2 H-ALG (TL%=81)	4.034	-4.059 to 12.13	No	ns	0.3884			
A: 0.2 L-ALG (TL%=81) vs. C: 0.4 L-ALG (TL%=34)	-13.78	-21.88 to -5.690	Yes	**	0.0043			
A: 0.2 L-ALG (TL%=81) vs. D: 0.4 H-ALG (TL%=34)	-23.82	-31.91 to -15.72	Yes	***	0.0002			
B: 0.2 H-ALG (TL%=81) vs. C: 0.4 L-ALG (TL%=34)	-17.82	-25.91 to -9.724	Yes	**	0.0011			
B: 0.2 H-ALG (TL%=81) vs. D: 0.4 H-ALG (TL%=34)	-27.85	-35.94 to -19.76	Yes	****	<0.0001			
C: 0.4 L-ALG (TL%=34) vs. D: 0.4 H-ALG (TL%=34)	-10.03	-18.13 to -1.940	Yes	*	0.0200			
Test details	Mean 1	Mean 2	Mean Diff.	SE of diff.	N1	N2	q	DF
A: 0.2 L-ALG (TL%=81) vs. B: 0.2 H-ALG (TL%=81)	15.26	11.22	4.034	2.338	3	3	2.440	6.000
A: 0.2 L-ALG (TL%=81) vs. C: 0.4 L-ALG (TL%=34)	15.26	29.04	-13.78	2.338	3	3	8.338	6.000
A: 0.2 L-ALG (TL%=81) vs. D: 0.4 H-ALG (TL%=34)	15.26	39.07	-23.82	2.338	3	3	14.41	6.000
B: 0.2 H-ALG (TL%=81) vs. C: 0.4 L-ALG (TL%=34)	11.22	29.04	-17.82	2.338	3	3	10.78	6.000
B: 0.2 H-ALG (TL%=81) vs. D: 0.4 H-ALG (TL%=34)	11.22	39.07	-27.85	2.338	3	3	16.85	6.000
C: 0.4 L-ALG (TL%=34) vs. D: 0.4 H-ALG (TL%=34)	29.04	39.07	-10.03	2.338	3	3	6.069	6.000

Table S11. Hydrodynamic diameter and Polydispersity index (PDI) of acid-gelled alginate nanoparticles without 5-FU loading (blank) in comparison with Ca+2-crosslinked alginate nanoparticles without 5-FU loading (blank) at pH 4.5.

**Nanoparticle**	Ca^{+2}-ALG NPs (0.4w/v%Alg.H) /10m MCaCl2 (Blank)	H^{+}-ALG NPs (0.4w/v%Alg.H)/ (Blank)
**Size (nm)**	978.7	402.8
**Size (nm)**	928.7	372.1
**Size (nm)**	1393	387.9

Table Analyzed	Copy of size
Column B	H^{+}-ALG NPs (0.4w/v%Alg.H)/ (Blank)
vs.	vs.
Column A	Ca^{+2}-ALG NPs (0.4w/v%Alg.H) /10m $MCaCl_2$ (Blank)
Unpaired t test	
P value	0.0084
P value summary	**
Significantly different (P < 0.05)?	Yes
One- or two-tailed P value?	Two-tailed
t, df	t=4.834, df=4
How big is the difference?	
Mean of column A	1100
Mean of column B	387.6
Difference between means (B - A) ± SEM	-712.5 ± 147.4
95% confidence interval	-1122 to -303.3
R squared (eta squared)	0.8538
F test to compare variances	
F, DFn, Dfd	275.6, 2, 2

**P value**	**0.0072**
**P value summary**	**
**Significantly different (P < 0.05)?**	**Yes**
Data analyzed	
Sample size, column A	3
Sample size, column B	3

**Nanoparticle**	Ca^{+2}-ALG NPs (0.4w/v%Alg.H) /10m MCaCl2 (Blank)	H^{+}-ALG NPs (0.4w/v%Alg.H)/ (Blank)
**PDI**	0.306	0.3761
**PDI**	0.2474	0.396
**PDI**	0.7002	0.3819

Table Analyzed	Copy of pdi
Column B	H^{+}-ALG NPs (0.4w/v%Alg.H)/ (Blank)
vs.	vs.
Column A	Ca^{+2}-ALG NPs (0.4w/v%Alg.H) /10m $MCaCl_2$ (Blank)
Unpaired t test	
P value	0.8270
P value summary	ns
Significantly different (P < 0.05)?	No
One- or two-tailed P value?	Two-tailed
t, df	t=0.2333, df=4
How big is the difference?	
Mean of column A	0.4179
Mean of column B	0.3847
Difference between means (B - A) ± SEM	-0.03320 ± 0.1423

95% confidence interval	-0.4283 to 0.3619
R squared (eta squared)	0.01343
F test to compare variances	
F, DFn, Dfd	579.0, 2, 2
**P value**	**0.0034**
**P value summary**	**
**Significantly different (P < 0.05)?**	**Yes**
Data analyzed	
Sample size, column A	3
Sample size, column B	3

Table S12. Hydrodynamic diameter and Polydispersity index (PDI) of acid-gelled alginate nanoparticles without 5-FU loading (blank) in comparison with Ca+2-crosslinked alginate nanoparticles without 5-FU loading (blank) at pH 10.

**Nanoparticle**	Ca^{+2}-ALG NPs (0.4w/v%Alg.H) /10m MCaCl2 (Blank)	H^{+}-ALG NPs (0.4w/v%Alg.H)/ (Blank)
**Size (nm)**	289.7	313.1
**Size (nm)**	334.9	301.7
**Size (nm)**	275.7	303.3

Table Analyzed	Copy of size
Column B	H^{+}-ALG NPs/2M HCL (0.4w/v%Alg.H)/ (Blank)
vs.	vs.
Column A	Ca^{+2}-ALG NPs/10m $MCaCl_2$ (0.4w/v%Alg.H) (Blank)
Unpaired t test	
P value	0.7610
P value summary	ns
Significantly different (P < 0.05)?	No
One- or two-tailed P value?	Two-tailed
t, df	t=0.3257, df=4
How big is the difference?	
Mean of column A	300.1
Mean of column B	306.0
Difference between means (B - A) ± SEM	5.933 ± 18.22
95% confidence interval	-44.64 to 56.51
R squared (eta squared)	0.02584
F test to compare variances	
F, DFn, Dfd	25.13, 2, 2

**P value**	**0.0765**
**P value summary**	**ns**
**Significantly different (P < 0.05)?**	**No**

**Nanoparticle**	Ca^{+2}-ALG NPs (0.4w/v%Alg.H) /10m MCaCl2 (Blank)	H^{+}-ALG NPs (0.4w/v%Alg.H)/ (Blank)
**PDI**	0.306	0.3761
**PDI**	0.2474	0.396
**PDI**	0.7002	0.3819

Table Analyzed	Copy of pdi
Column B	H^{+}-ALG NPs/2M HCL (0.4w/v%Alg.H)/ (Blank)
vs.	vs.
Column A	Ca^{+2}-ALG NPs/10m $MCaCl_2$ (0.4w/v%Alg.H) (Blank)
Unpaired t test	
P value	0.0073
P value summary	**
Significantly different (P < 0.05)?	Yes
One- or two-tailed P value?	Two-tailed
t, df	t=5.045, df=4
How big is the difference?	
Mean of column A	0.5082
Mean of column B	0.3399
Difference between means (B - A) ± SEM	-0.1683 ± 0.03336
95% confidence interval	-0.2609 to -0.07568

R squared (eta squared)	0.8642
F test to compare variances	
F, DFn, Dfd	7.991, 2, 2
**P value**	**0.2225**
**P value summary**	**ns**
**Significantly different (P < 0.05)?**	**No**
Data analyzed	
Sample size, column A	3
Sample size, column B	3
